# Combined effects of functional overload and denervation on skeletal muscle mass and its regulatory proteins in mice

**DOI:** 10.14814/phy2.15689

**Published:** 2023-05-09

**Authors:** Kazuki Uemichi, Takanaga Shirai, Tohru Takemasa

**Affiliations:** ^1^ Graduate School of Comprehensive Human Sciences University of Tsukuba Ibaraki Japan; ^2^ Faculty of Health and Sport Sciences University of Tsukuba Ibaraki Japan

**Keywords:** denervation, functional overload, skeletal muscle

## Abstract

Skeletal muscle is a highly pliable tissue and various adaptations such as muscle hypertrophy or atrophy are induced by overloading or disuse, respectively. However, the combined effect of overloading and disuse on the quantitative adaptation of skeletal muscle is unknown. Thus, the aim of this study was to investigate the effects of the combined stimuli of overloading and disuse on mouse skeletal muscle mass and the expression of regulatory factors for muscle protein anabolism or catabolism. Male mice from the Institute Cancer Research were subjected to denervation concomitant with unilateral functional overload or functional overload concomitant with unilateral denervation. Disuse and functional overload were induced by sciatic nerve transection (denervation) and synergist ablation, respectively, and the plantaris muscle was harvested 14 days after the operation. Our results showed that denervation attenuated functional overload‐induced muscle hypertrophy and functional overload partially ameliorated the denervation‐induced muscle atrophy. P70S6K phosphorylation, an indicator of mechanistic target of rapamycin complex 1 (mTORC1) activation, was not increased by unilateral functional overload in denervated muscles or by unilateral denervation in functional overloaded muscles. Denervation did not affect the increase of LC3‐II, a marker of autophagy activation, and ubiquitinated protein expression upon unilateral functional overload. Also, functional overload did not affect ubiquitinated protein expression during unilateral denervation. Thus, our findings suggest that functional overload‐induced muscle hypertrophy or denervation‐induced muscle atrophy was attenuated by the combined stimuli of overload and denervation.

## Graphical abstract

The aim of this study was to investigate the effects of the combined stimuli of overloading and disuse on mouse skeletal muscle mass and the expression of regulatory factors for muscle protein anabolism or catabolism. The mice were subjected to denervation concomitant with unilateral functional overload or functional overload concomitant with unilateral denervation.

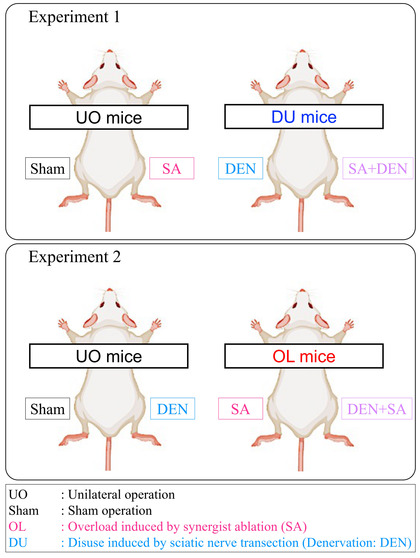

## 1 INTRODUCTION

Skeletal muscle is known to have a high degree of quantitative plasticity, such as hypertrophy in response to mechanical loading (e.g., functional overload, resistance exercise—Boone et al., 2015; McCarthy et al., 2011), and atrophy in response to unloading (e.g., space flight, inactivity—Hayashi et al., 2021; Zhang et al., 2018). Skeletal muscle mass is largely dependent on protein metabolism, which is the balance between the synthesis and degradation of muscle proteins (Tipton & Wolfe, 2001).

Surgical synergist ablation‐induced functional overload is an experimental model that has classically been used to investigate the response of skeletal muscle hypertrophy and its molecular mechanisms in rodents (Bodine et al., 2001). This surgical operation induces a compensatory hypertrophy in the plantaris muscle by ablation of the synergist muscles, gastrocnemius and soleus (Fujimaki et al., 2016; Shirai et al., 2022; Uemichi et al., 2021). Previous studies have reported that functional overload activated mechanistic target of rapamycin complex 1 (mTORC1) signaling pathway, a key regulatory mechanism for muscle protein synthesis, and induced hypertrophy (Goodman et al., 2011; Miyazaki et al., 2011). Conversely, skeletal muscle inactivity, such as disuse, immobilization, and space flight, dramatically decreased muscle mass (Brook et al., 2022; Hayashi et al., 2021; Zhang et al., 2018). Denervation (DEN) is a commonly employed as an atrophy model for rodent skeletal muscle (Quy et al., 2013; You et al., 2021). This surgical operation removes the innervation to the lower legs by sciatic nerve transection (Machida et al., 2012; Tamura et al., 2015). Mice with transected sciatic nerve legs cannot plantar flex the ankle joint, but can flex the hip joint, so they walk with only their toes on the ground, not the whole sole. It seems gastrocnemius, plantaris, and soleus muscles cannot contract, while mechanical overload from walking and grounding is still present in the legs of mice. Previous studies have reported that the DEN‐induced atrophy was marked by enhanced ubiquitin‐proteasome and autophagy systems that are the main proteolytic mechanisms in skeletal muscle (Kitaoka et al., 2016). Furthermore, synergist ablation induces muscle hypertrophy and DEN induces muscle atrophy, but the molecular signals involved in both muscle protein synthesis and degradation are upregulated by synergist ablation or DEN (Moriya & Miyazaki, 2018; Tang et al., 2014).

Recently, researchers have shown that percutaneous electrical stimulation‐induced muscle contraction partially ameliorated muscle atrophy associated with hindlimb suspension (Kotani et al., 2022). In addition, mouse skeletal muscle atrophy associated with hindlimb suspension demonstrated a protein synthesis response to electrical stimulation‐induced resistance exercise after the period of suspension comparable to that of the non‐atrophied muscle (Ato et al., 2020). Thus, it is suggested that overload stimulation of skeletal muscle is effective to maintain skeletal muscle mass even during inactivity. However, the effects of concurrent overloading and inactivity on skeletal muscle hypertrophic or atrophic responses and the expression of their regulatory signaling molecules are unknown. Therefore, we aimed to determine the effects of the combined functional overload and DEN on muscle hypertrophy or atrophy.

## 2 METHODS

### 2.1 Experimental animals

**FIGURE 1:**
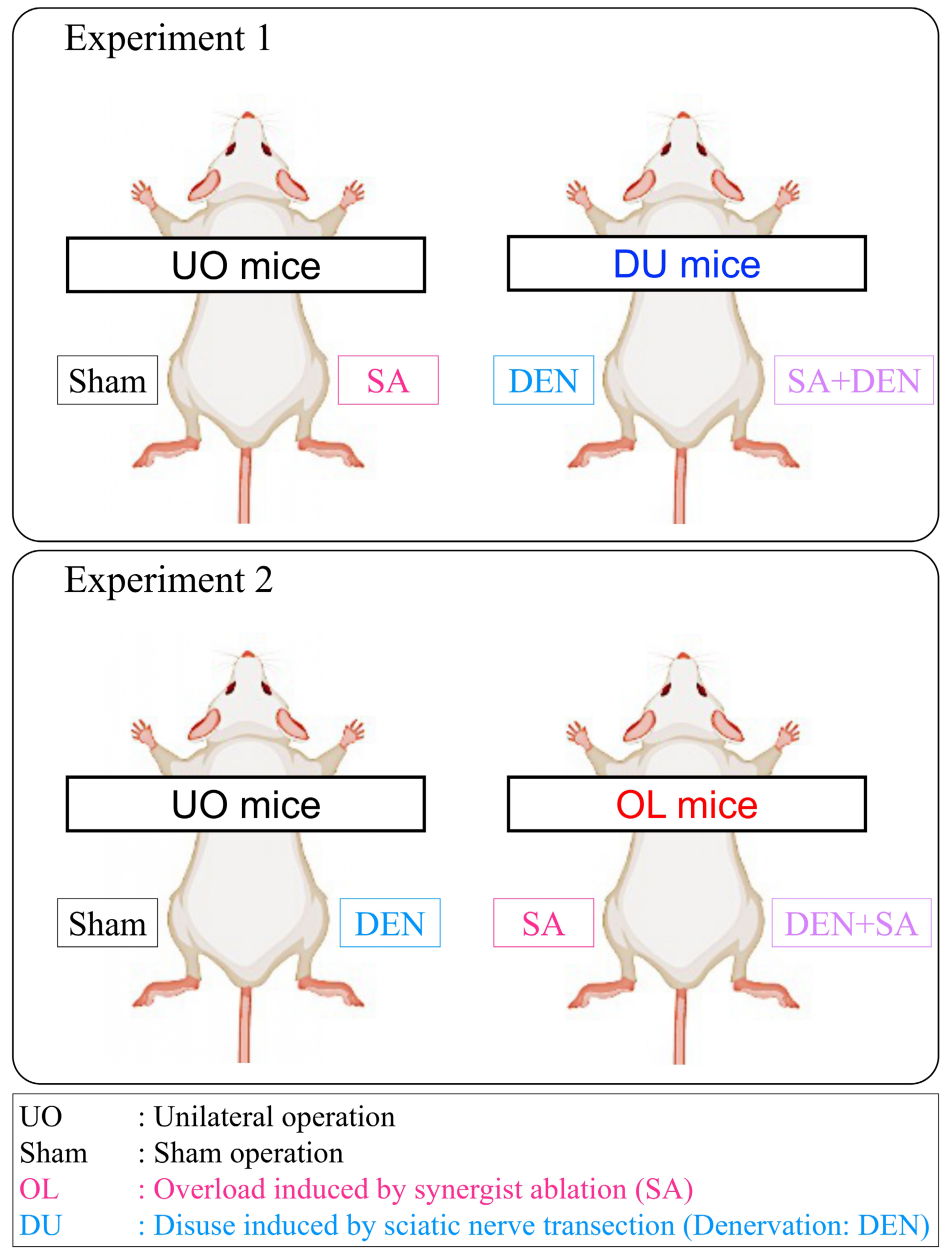
Graphical abstract of the experimental protocol. In Experiment 1, the left plantaris of unilateral operated (UO) mice were sham‐operated and the right plantaris were subjected to synergist ablation (SA)‐induced functional overload, while the left plantaris of disuse (DU) mice was subjected to sciatic nerve transection‐induced denervation and the right plantaris was subjected to both functional overload and denervation (upper side of the panel). In Experiment 2, the left plantaris of UO mice was sham‐operated and the right plantaris was subjected to sciatic nerve transection‐induced denervation, while the left plantaris of OL mice was subjected to SA‐induced functional overload and the right plantaris was subjected to both denervation and functional overload (lower side of the panel).

Seven‐week‐old male Institute of Cancer Research mice (Tokyo Laboratory Animals Science Co.) were purchased from Oriental Yeast Co. Ltd. (Tokyo, Japan) and housed at 22°C ± 2°C and 55% ± 5% humidity controlled holding facilities under a 12 h light/dark cycle, with ad libitum access to food and water. Experimental animals were classified into unilateral operated (UO) or disuse (DU) mice (Experiment 1) and UO or overloaded (OL) mice (Experiment 2). The UO mice were subjected to functional overload by synergist ablation (SA) in Experiment 1 or DEN by sciatic nerve transection in Experiment 2 in the right leg and sham‐operated in the left leg. The DU mice were subjected to sciatic nerve transection in both legs and concomitant SA in the right leg. The OL mice were subjected to SA in both legs and concomitant DEN in the right legs. The graphical abstract of each experimental protocol is shown in Figure 1.

### 2.2 SA surgery

SA surgeries were performed under anesthesia with isoflurane (2.0% isoflurane in air) inhalation as previously described (Egner et al., 2016; Moriya & Miyazaki, 2018). This in vivo model induces hypertrophy of the plantaris muscle through functional overload, resulting from the surgical dissection of synergist muscles (gastrocnemius and soleus). The posterior aspects of the lower legs were incised, and the gastrocnemius and soleus muscles were removed using forceps and scissors. Fourteen days after the surgery, the mice were euthanized by cervical dislocation, and the plantaris muscle was excised, weighed, frozen in liquid nitrogen, and stored at −80°C until further analysis.

### 2.3 DEN surgery

DEN surgery was performed with sciatic nerve transection under anesthesia with isoflurane inhalation (2.0%–3.0% isoflurane in air) after 7 days of acclimation, as described (MacDonald et al., 2014). The mice were anesthetized in a prone position, and the sciatic nerve of the hindlimb was exposed. At least 5.0 mm of the sciatic nerve was transected using small operating scissors, and the skin was closed with surgical adhesive. Although the plantaris, gastrocnemius, and soleus muscles of mice with transected sciatic nerve are denervated and unable to plantar flex the ankle joint, hip flexion is possible, and the mice were allowed to move freely within the cage. Denervated mice did not walk using the entire surface of the sole, but only the toes. Fourteen days after the surgery, the mice were euthanized by cervical dislocation. The plantaris muscle was excised, weighed, frozen in liquid nitrogen, and stored at −80°C until analysis.

### 2.4 Western blotting

Isolated muscle samples were immediately frozen in liquid nitrogen and total muscle protein was extracted from a homogenize solution containing 50 mM of HEPES (pH: 7.6), 150 mM NaCl, 10 mM EDTA, 10 mM Na_4_P_2_O_7_, 10 mM NaF, 2 mM Na_3_VO_4_, 1% (v/v) NP‐40, 1% (v/v) Na‐deoxycholate, 0.2% (w/v) sodium dodecyl sulfate (SDS), and 1% (v/v) complete protease inhibitor cocktail. Protein concentrations were measured using the Protein Assay Bicinchoninate Kit (Nacalai Tesque Inc.). Prior to SDS‐PAGE, an aliquot of the extracted protein solution was mixed with an equal volume of sample loading buffer containing 1% (v/v) 2‐mercaptoethanol, 4% (w/v) SDS, 125 mM Tris–HCl (pH: 6.8), 10% (w/v) sucrose, and 0.01% (w/v) bromophenol blue. Five micrograms of protein was separated by SDS‐PAGE and transferred to an Immuno‐Blot PVDF membrane (Bio‐Rad Laboratories). The blot was blocked using Blocking One (Nacalai Tesque Inc.) for 1 h at 22 ± 2°C and incubated with primary antibodies in Western Blot Immuno Booster (Takara Bio) or Can Get Signal solution (TOYOBO) overnight at 4°C. After overnight incubation, the membranes were incubated with horseradish peroxidase‐conjugated secondary antibodies (#7074P2 or #7076S, Cell Signaling Technology) for 60 min at 22 ± 2°C. Signals were detected using ImmunoStar Zeta or LD (FUJIFILM Wako Pure Chemical Co.), quantified using C‐Digit (LI‐COR Biosciences), and expressed as arbitrary units. Coomassie brilliant blue staining was used to verify consistent loading.

### 2.5 Primary antibodies for western blotting

The following primary antibodies were used for western blotting: 1:1000 anti‐Akt (#9272S), 1:1000 anti‐p‐Akt (Thr308, #13038S), 1:1000 anti‐p‐Akt (Ser473, #9271S), 1:1000 anti‐p‐FoxO1 (Ser256, 9461S), 1:1000 anti‐FoxO1 (#2880S), 1:1000 anti‐LC3 (#4108S), 1:1000 anti‐eIF4E‐binding protein 1 (4E‐BP1) (#9452), 1:1000 anti‐p‐4E‐BP1 (Thr37/46, #2855S), 1:1000 anti‐p‐4E‐BP1 (Ser65), 1:1000 anti‐P70S6K (#9202), 1:1000 anti‐p‐P70S6K (Thr389, #9205), 1:1000 anti‐p‐P70S6K (Thr421/Ser424, #9204S), 1:1000 anti‐rpS6 (#2217), 1:1000 anti‐p‐rpS6 (Ser240/244, #5364P), 1:1000 anti‐p‐rpS6 (Ser235/236, #4858S). These antibodies were purchased from Cell Signaling Technology. 1:1000 anti‐ubiquitin (SC‐8017) was purchased from Santa Cruz Biotechnology. 1:1000 anti‐p62/SQSTM (lot. 022) was purchased from Medical & Biological Laboratories.

### 2.6 Statistical analyses

Data are shown as mean ± standard deviation (SD). We performed unpaired Student's *t*‐tests or two‐way analysis of variance followed by the Tukey's post hoc test. GraphPad Prism 8 (GraphPad, Inc.) was used for all statistical calculations, and significance was set at *p* < 0.05 in all instances.

## 3 RESULTS

### 3.1 Body mass and skeletal muscle wet weight

#### 3.1.1 Experiment 1

**FIGURE 2:**
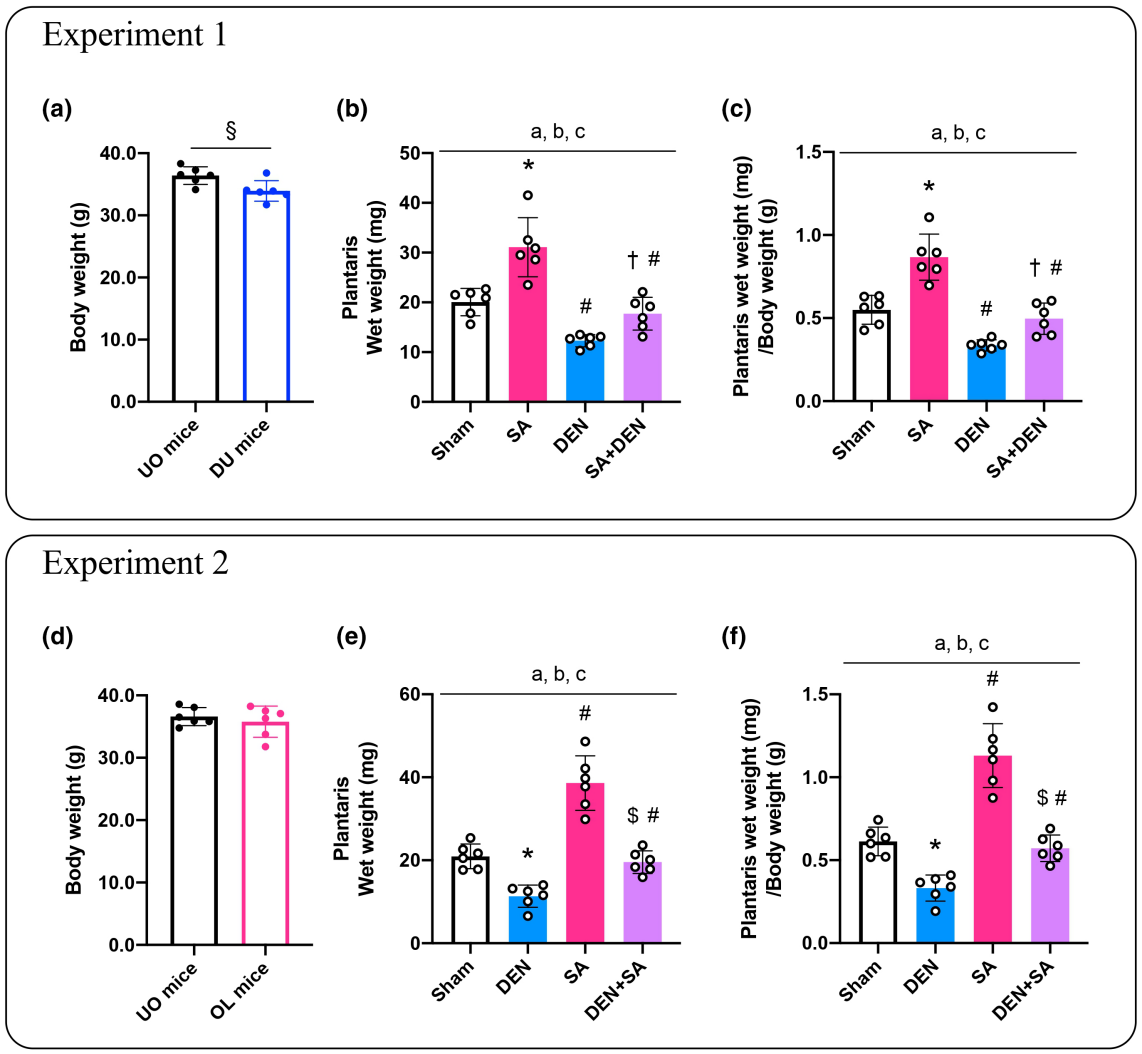
Body weight changes in disuse (DU) mice (a). Effects of lateral denervation on overload‐induced changes in plantaris wet weight (b), and plantaris wet weight per body mass (c). The change of body weight in overloaded (OL) mice (d). Effects of lateral overload on denervation‐induced changes in plantaris wet weight (e), and plantaris wet weight per body mass (f). Values are presented as mean ± standard deviation (SD). ^†^ *p* < 0.05 versus control. ^a^Main effect of overload (*p* < 0.05); ^b^main effect of denervation (*p* < 0.05); ^c^interaction of overload and denervation (*p* < 0.05); ^§^ *p* < 0.05 versus UO mice; *p < 0.05 vs. sham‐operated legs in UO mice; ^#^ *p* < 0.05 versus corresponding legs in UO mice; ^†^ *p* < 0.05 versus only denervated legs in DU mice; ^$^ *p* < 0.05 versus only overloaded legs in OL mice. *n* = 5–6 per condition and group.

The body weight in DU mice was significantly reduced compared with UO mice (*p* = 0.019, Figure 2a). Functional overload significantly increased the plantaris muscle wet weight in UO and DU mice compared with the sham‐operated or only denervated muscle (*p* < 0.001, *p* = 0.001, respectively; Figure 2b). In addition, DU mice had significantly lower plantaris muscle wet weight in both the only denervated and overloaded plus denervated muscles compared with that of the UO mice (*p* = 0.001, *p* < 0.001, respectively; Figure 2b). Similarly, the plantaris wet weight corrected by body weight was significantly higher in the overloaded muscle compared with the sham‐operated muscle in UO mice and compared with the only denervated muscle in DU mice (*p* < 0.001, *p* = 0.001, respectively; Figure 2c). DU mice had significantly lower plantaris wet weight than sham‐operated or overloaded muscle in UO mice (*p* = 0.001, *p* < 0.001, respectively; Figure 2c).

#### 3.1.2 Experiment 2

No significant change in body weight was observed between UO and OL mice (Figure 2d). Unilateral DEN significantly reduced the plantaris wet weight in UO and OL mice compared with the sham‐operated or only overloaded muscle (*p* = 0.001, *p* < 0.001, respectively; Figure 2e). In addition, OL mice had a significantly higher plantaris wet weight than UO mice with or without DEN (*p* = 0.001, *p* = 0.040, respectively; Figure 2e). Similarly, unilateral DEN significantly reduced the plantaris wet weight corrected by body weight compared with that of the non‐denervated muscles regardless of mice type (UO mice: *p* = 0.001; OL mice: *p* < 0.001; Figure 2f). In OL mice, plantaris corrected by body weight was significantly higher than UO mice in each leg (sham: *p* < 0.001; DEN: *p* = 0.017; Figure 2f).

### 3.2 Signaling related to muscle protein synthesis

#### 3.2.1 Protein kinase B (Akt) /P70 ribosomal S6 kinase (P70S6K) signaling; Experiment 1

**FIGURE 3:**
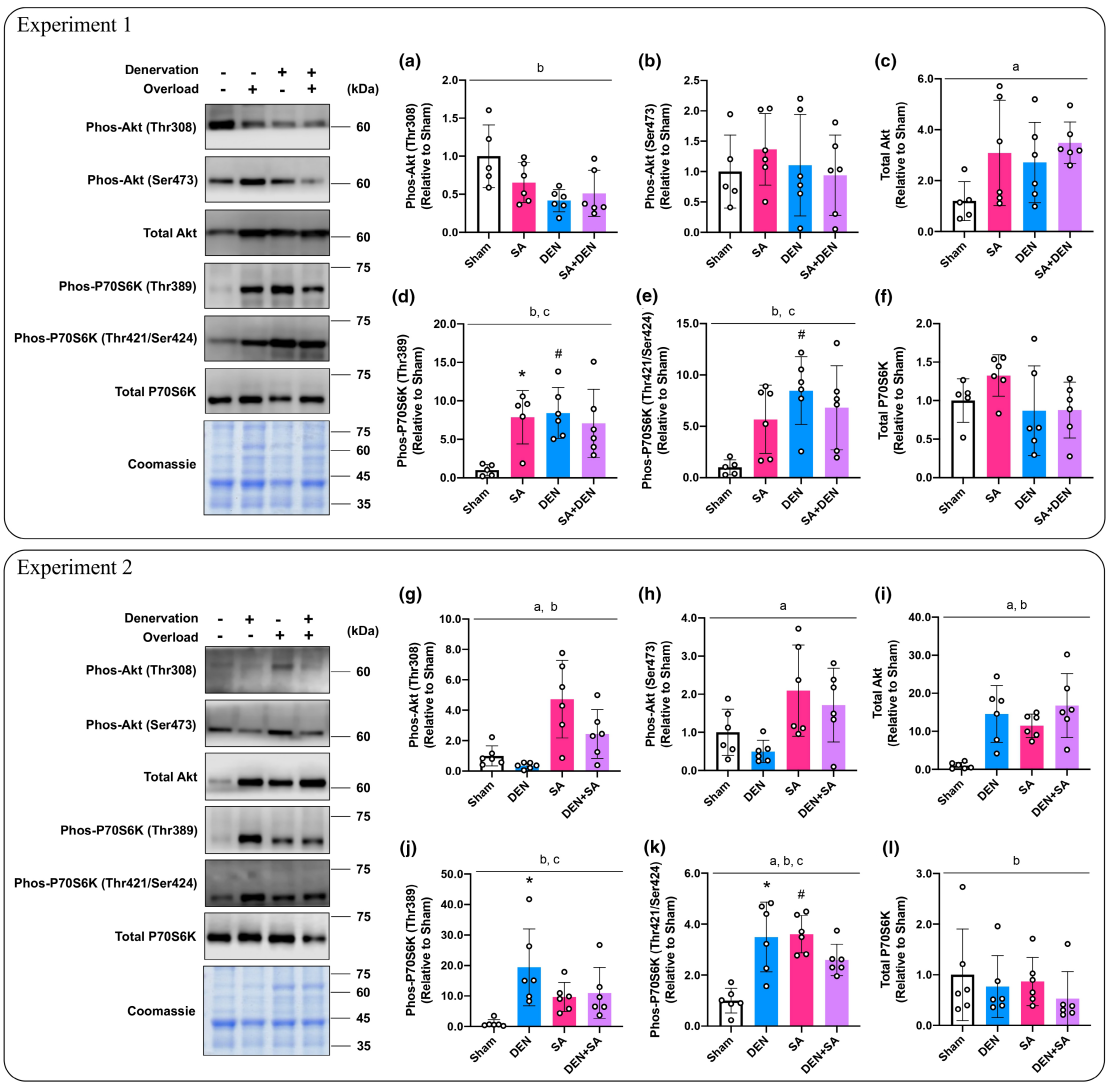
Effects of lateral denervation on overload‐induced changes of Akt (a–c) and P70S6K (d–f). Effects of lateral overload on denervation‐induced changes of Akt (g–i) and P70S6K (j–l). In each experiment, the same coomassie‐stained images were presented as a representative image. Protein phosphorylation and total content are expressed relative to the sham‐operated legs in the unilateral operated (UO) mice. Values are presented as mean ± standard deviation (SD). ^a^Main effect of overload (*p* < 0.05); ^b^main effect of denervation (*p* < 0.05); ^c^interaction of overload and denervation (*p* < 0.05); *p < 0.05 versus sham‐operated legs in UO mice; ^#^ *p* < 0.05 versus corresponding legs in UO mice. *n* = 5–6 per condition and group.

We evaluated the effects of concurrent SA and DEN on Akt/P70S6K signaling expression, which regulates muscle protein synthesis (Bodine et al., 2001; Castets et al., 2019). In the experiment with DU mice, we observed that DEN had a significant effect on Thr308 phosphorylation of Akt (*p* = 0.007, Figure 3a) but had no effect on Ser473 phosphorylation (Figure 3b). An effect of SA was observed on the total Akt content (*p* = 0.039, Figure 3c). P70S6K phosphorylation at Thr389 was significantly increased by SA or DEN alone compared with the sham‐operated muscle (*p* = 0.021, *p* = 0.009, respectively, Figure 3d), but no increase by SA was observed in DU mice. Similarly, the P70S6K phosphorylation at Thr421/Ser424 was significantly increased by DEN alone compared with the sham‐operated muscle (*p* = 0.005, Figure 3e), but no combined effect of SA and DEN was observed. We observed no changes in total P70S6K content (Figure 3f).

#### 3.2.2 Akt/P70S6K signaling; Experiment 2

In the experiment with OL mice, the additive effect of SA and DEN on Akt phosphorylation at Thr308 was confirmed (effect of SA: *p* = 0.002; effect of DEN: *p* = 0.022; Figure 3g), but only SA had a significant effect on Ser473 phosphorylation (*p* = 0.011, Figure 3h). The additive effects of SA and DEN were confirmed for total Akt content (effect of SA: *p* = 0.015; effect of DEN: *p* < 0.001; Figure 3i). In the UO mice, DEN significantly increased Thr389 and the Thr421/Ser424 phosphorylation of P70S6K (*p* = 0.003, *p* < 0.001, respectively, Figure 3j,k). Furthermore, SA alone significantly increased the Thr421/Ser424 phosphorylation of P70S6K compared with the sham‐operated muscle (*p* < 0.001, Figure 3k), whereas an increase by DEN was not observed in the OL mice. The significant effect of DEN on total P70S6K content was confirmed (*p* = 0.005, Figure 3l).

#### 3.2.3 Ribosomal protein S6 (RpS6)/eukaryotic initiation factor 4E‐binding protein 1 (4E‐BP1) signaling; Experiment 1

**FIGURE 4:**
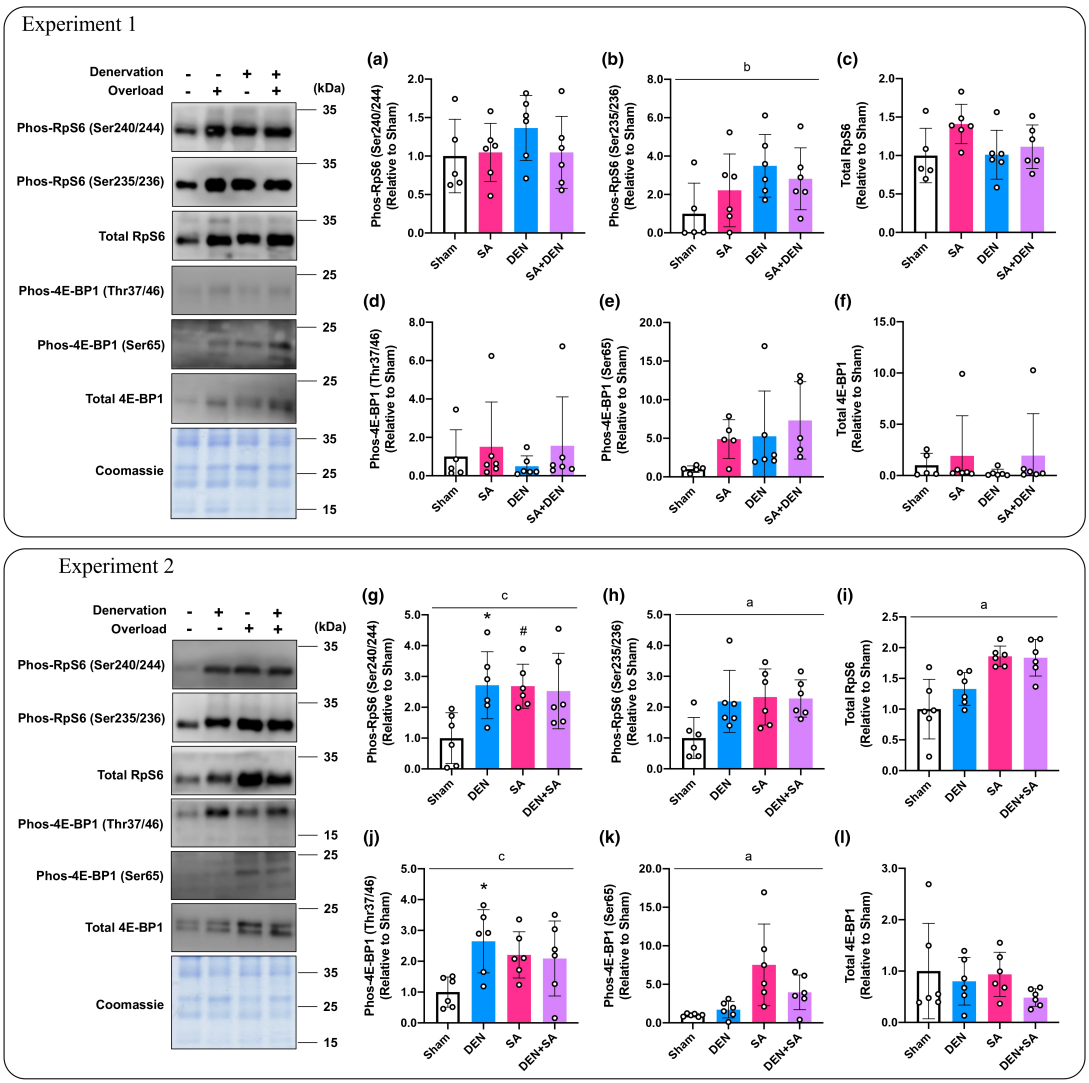
Effects of lateral denervation on overload‐induced changes of RpS6 (a–c) and 4E‐BP1 (d–f). Effects of lateral overload on denervation‐induced changes of RpS6 (g–i) and 4E‐BP1 (j–l). In each experiment, the same coomassie‐stained images were presented as a representative image. Protein phosphorylation and total content are expressed relative to the sham‐operated legs in the unilateral operated (UO) mice. Values are presented as mean ± standard deviation (SD). ^a^Main effect of overload (*p* < 0.05); ^b^main effect of denervation (*p* < 0.05); ^c^interaction of overload and denervation (*p* < 0.05); *p < 0.05 vs. sham‐operated legs in UO mice; ^#^ *p* < 0.05 versus corresponding legs in UO mice. *n* = 5–6 per condition and group.

RpS6 and 4E‐BP1 are representative molecules that regulate translational capacity and initiation, respectively (Willett et al., 2009). In experiments with DU mice, the main effect of DEN was only observed in RpS6 phosphorylation at the Ser235/236 site (*p* = 0.041, Figure 4b), while Ser240/244 phosphorylation and total protein content were unchanged (Figure 4a,c). We found no significant changes in the phosphorylation of 4E‐BP1 and total protein content (Figure 4d–f).

#### 3.2.4 RpS6/4E‐BP1 signaling; Experiment 2

In muscles subjected to only DEN or SA, RpS6 phosphorylation (Ser240/244) was significantly increased compared with the sham‐operated muscle in the UO mice (*p* = 0.031, *p* = 0.035, respectively; Figure 4g). The significant effect of SA was confirmed in the phosphorylation of RpS6 and total protein content (*p* = 0.044, *p* < 0.001, respectively; Figure 4h,i). DEN significantly increased 4E‐BP1 Thr37/46 phosphorylation in UO mice (*p* = 0.009, Figure 4j), but not in OL mice. The effect of SA on the phosphorylation of 4E‐BP1 Ser65 was confirmed (*p* = 0.006, Figure 4k,l) but no effect on total protein content was observed.

### 3.3 Signaling related to muscle protein degradation

#### 3.3.1 Ubiquitin‐proteasome system‐related signaling; experiment 1

**FIGURE 5:**
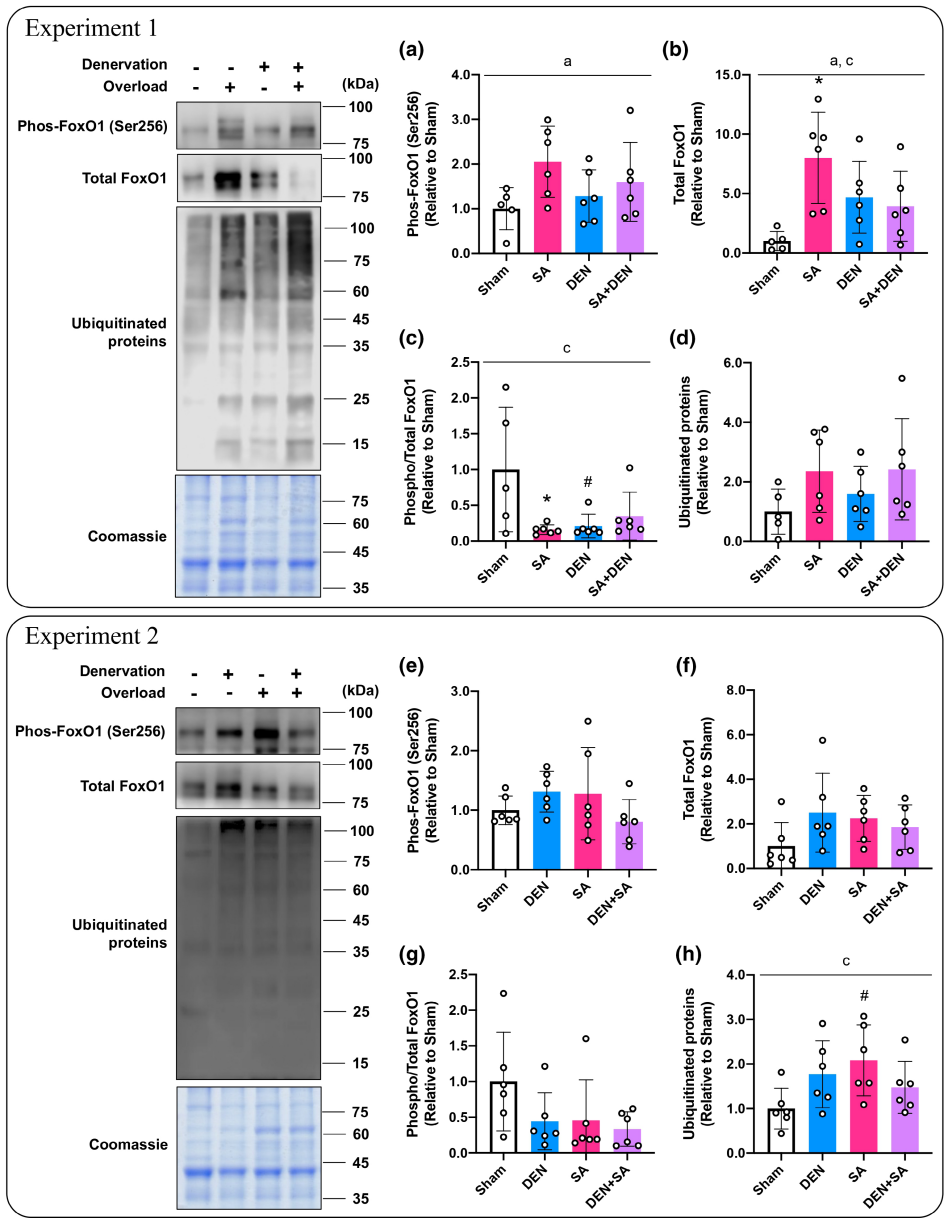
Effects of lateral denervation on overload‐induced changes of FoxO1 (a–c) and ubiquitinated proteins (d). Effects of lateral overload on denervation‐induced changes of FoxO1 (e–g) and ubiquitinated proteins (h). In each experiment, the same coomassie‐stained images were presented as a representative image. Protein phosphorylation, total content, and phosphor/total ratios are expressed relative to the sham‐operated legs in the unilateral operated (UO) mice. Values are presented as mean ± standard deviation (SD). ^a^Main effect of overload (*p* < 0.05); ^b^main effect of denervation (*p* < 0.05); ^c^interaction of overload and denervation (*p* < 0.05); *p < 0.05 versus sham‐operated legs in UO mice; ^#^ *p* < 0.05 versus corresponding legs in UO mice. *n* = 5–6 per condition and group.

We measured the FoxO1 protein, which is a transcription factor of ubiquitin ligases and ubiquitinated proteins to evaluate muscle proteolytic response via the ubiquitin‐proteasome system. In the experiment with DU mice, the main effects of SA were confirmed in FoxO1 Ser256 phosphorylation and total protein content (*p* = 0.034, *p* = 0.024, respectively; Figure 5a,b). In UO mice, SA significantly increased the total FoxO1 protein compared with the sham‐operated muscle (*p* = 0.004, Figure 5b), and no changes were observed in DU mice. The phosphorylation/total ratio of FoxO1 was significantly reduced only in the synergist ablated or denervated muscle compared with the sham‐operated muscle in UO mice (*p* = 0.026, *p* = 0.039, respectively; Figure 5c). However, no changes in ubiquitinated proteins were noted (Figure 5d).

#### 3.3.2 Ubiquitin‐proteasome system‐related signaling; Experiment 2

In the experiment with OL mice, the Ser256 phosphorylation, total protein, and phosphorylation/total ratio of FoxO1 were unaltered (Figure 5e–g). On the other hand, in OL mice, ubiquitinated proteins were significantly higher in synergist ablated muscle than in sham‐operated muscle in UO mice (*p* = 0.045, Figure 5h).

#### 3.3.3 Autophagy system‐related signaling; Experiment 1

**FIGURE 6:**
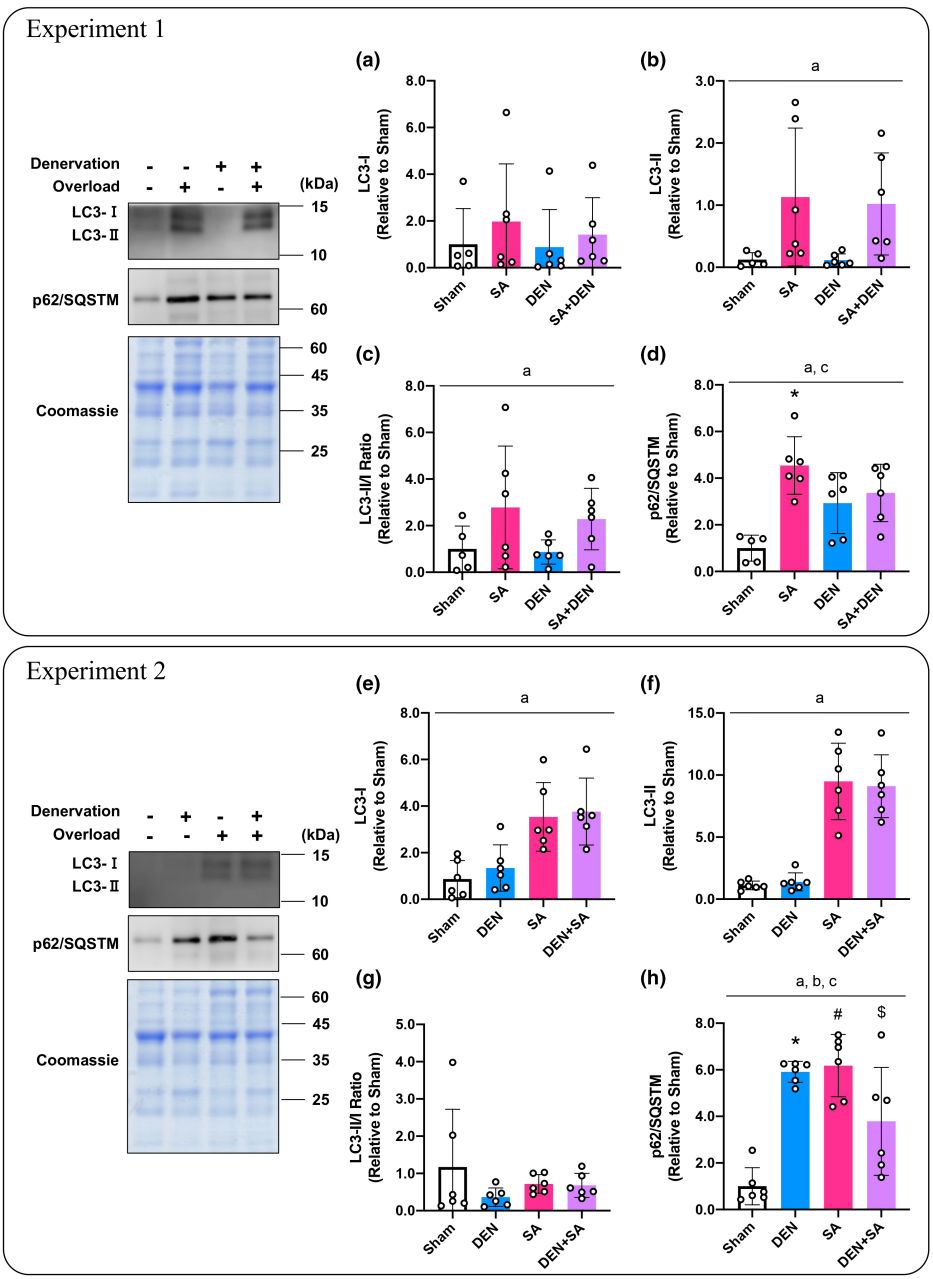
Effects of lateral denervation on overload‐induced changes of LC3‐I (a), LC3‐II (b), LC3‐II/I ratio (c), and p62/SQSTM (d). Effects of lateral overload on denervation‐induced changes of LC3‐I (e), LC3‐II (f), LC3‐II/I ratio (g), and p62/SQSTM (h). In each experiment, the same coomassie‐stained images were presented as a representative image. Protein content or ratios are expressed relative to the sham‐operated legs in the unilateral operated (UO) mice. Values are presented as mean ± standard deviation (SD). ^a^Main effect of overload (*p* < 0.05); ^b^main effect of denervation (*p* < 0.05); ^c^interaction of overload and denervation (*p* < 0.05); *p < 0.05 versus sham‐operated legs in UO mice; ^#^ *P* < 0.05 versus corresponding legs in UO mice. ^$^ *p* < 0.05 versus only overloaded legs in overloaded (OL) mice. *n* = 5–6 per condition and group.

To evaluate the autophagy response, we measured LC3 (a marker of autophagosome formation) and p62/SQSTM (a marker of selective autophagy) protein content. In the experiment with DU mice, LC3‐I was unchanged, but the significant effect of SA alone on the LC3‐II and LC3‐II/I ratio was confirmed (*p* = 0.004, *p* = 0.027, respectively; Figure 6b,c). In addition, in UO mice, SA significantly increased the p62/SQSTM compared with the sham‐operated muscle (*p* < 0.001, Figure 6d), but not in DU mice.

#### 3.3.4 Autophagy system‐related signaling; experiment 2

Experiments with OL mice confirmed the effect of SA on LC3‐I and LC3‐II content (*p* = 0.002, *p* < 0.001, respectively; Figure 6e,f). No change in the LC3‐II/I ratio was observed (Figure 6g). Furthermore, p62/SQSTM levels were significantly higher in denervated muscle in UO mice and only in overloaded muscle of OL mice than in sham‐operated muscle in UO mice (*p* < 0.001, *p* < 0.001, respectively; Figure 6h). However, denervated plus synergist ablated muscles in OL mice were significantly lower than the overloaded muscles in the same group (*p* = 0.037, Figure 6h).

## 4 DISCUSSION

The present study investigated the effects of concurrent SA and DEN on muscle hypertrophy or atrophy and protein anabolism or catabolism‐related signaling activity. The main findings of the present study are: (1) In the muscle of DU mice, the activation of Akt/mTORC1 signaling molecules by SA did not occur and muscle mass gain was attenuated. Interestingly, the activation of LC3 by SA was comparable between DU and UO mice. (2) In OL mice, DEN‐induced atrophy was partially ameliorated, while the activation of mTORC1 downstream signaling (P70S6K, RpS6, and 4E‐BP1) in the denervated muscle was absent. Furthermore, ubiquitinated proteins and p62/SQSTM expression were suppressed in the denervated muscle of OL mice. These findings suggest that the concurrent loading of SA‐induced functional overload and DEN suppressed either muscle hypertrophic or atrophic responses.

Muscle protein synthesis via the mTORC1 pathway is facilitated by DEN and SA (Tabbaa et al., 2021; You et al., 2018). However, in the present study, the SA of DU mice or DEN of OL mice did not enhance the phosphorylation of P70S6K, RpS6, or 4E‐BP1. Activation of P70S6K by SA was reported to occur immediately and persist until 10–14 days after surgery (Miyazaki et al., 2011; Pérez‐Schindler et al., 2013). Similarly, activation of P70S6K and RpS6 by DEN was reported to occur on Day 2 and persist until Day 28 after sciatic nerve transection (Castets et al., 2019; Quy et al., 2013). Thus, it is suggested that the period of sustained activation of mTORC1 downstream molecules overlaps between SA and DEN; therefore, the signaling activation was not augmented by concurrent loading. Akt Ser473 phosphorylation is a well‐known downstream target of mTOR complex 2 (mTORC2), another protein complex that includes mTOR, and was activated by functional overload (Kido et al., 2021; Ogasawara et al., 2019). In the present study, Akt Ser473 phosphorylation was increased in OL mice, while it was unaffected by DEN. It was reported that the Akt Ser473 phosphorylation was not affected by DEN (Tang et al., 2014), which is consistent with the data of the present study. Insulin/phosphoinositide 3‐kinase signaling‐dependent mTORC2 localization to the ribosomes activates Akt (Smith et al., 2020; Zinzalla et al., 2011). Thus, it is suggested that increased translational efficiency via mTORC2/Akt signaling activation contributed to the attenuation of DEN‐induced atrophy in OL mice. In the present study, the increased RpS6 Ser235/236 phosphorylation and total protein content in OL mice were not affected by DEN. Ribosome biogenesis via mTORC1 signaling activation increased translational capacity and contributed to muscle protein synthesis (Chaillou et al., 2014). RpS6 is a protein component of the 40S ribosomal subunit and reflects ribosomal content as an indicator of protein synthesis (Ato & Ogasawara, 2021; Yi et al., 2021). Therefore, it is suggested that the increased translational capacity associated with functional overload contributed to the partial amelioration due to DEN. It should be noted that 47S‐pre rRNA and 18S+28S rRNA, which are indicators of ribosome biogenesis (Figueiredo et al., 2021; Kotani et al., 2021b) were not investigated in the present study.

Ubiquitin–proteasome system‐dependent muscle protein degradation was reported to increase in both functional overload and DEN (Koltai et al., 2017; Tang et al., 2014). However, we did not observe any enhanced effects of SA and DEN on FoxO1 phosphorylation or protein content and ubiquitinated protein levels. The results of this study indicate that SA or DEN alone promoted FoxO1 dephosphorylation and increased muscle protein degradation, which is in agreement with previous studies (Goodman et al., 2011; You et al., 2018). Akt activation negatively regulates FoxO1 and inhibits the increase in muscle protein degradation (Nishimura et al., 2022). In this study, the phosphorylation of Akt Thr308 was reduced in DU mice compared with UO mice, while FoxO1 phosphorylation was unchanged. In contrast, ubiquitinated protein expression was increased by SA alone, but not in the denervated muscle of OL mice compared with UO mice. Jun N‐terminus kinase (JNK) and STE20‐like protein kinase activation induced by oxidative and genotoxic stress causes the nuclear translocation of FoxO independent of Akt regulation (Hay, 2011). It has been reported that DEN increased the JNK phosphorylation in mouse muscle (Li et al., 2021). These findings suggest that FoxO‐mediated ubiquitin‐proteasome system‐dependent proteolysis in concurrent loaded muscle is independent of Akt regulation. LC3 is required for autophagosome formation and is a regulator of autophagy activation (Mizushima & Yoshimori, 2007). Previous studies have reported that LC3 activation is reduced by functional overload, whereas it is increased by DEN (MacDonald et al., 2014; Moriya & Miyazaki, 2018). However, in this study, LC3‐II content or LC3‐II/I ratio were increased by SA, whereas DEN had no effect. Thus, it is suggested that LC3 activation by SA exceeded the activation level during DEN in concurrently loaded muscle. Repeated electrical stimulation‐induced muscle contractions increased the LC3‐II content (Kotani et al., 2021a). The extent of contraction intensity in functional overloaded muscle is unclear, but it is suggested that chronic high‐intensity muscle contractions activate LC3 and promote autophagosome formation. In accordance with previous studies, SA or DEN alone increased the content of p62/SQSTM (Kitaoka et al., 2016; Moriya & Miyazaki, 2018), a marker of selective autophagy, whereas concurrent loading did not enhance the increase the level of p62/SQSTM. Therefore, LC3 activation was unaffected by concurrent loading of SA and DEN, suggesting that the removal of degradative substrates encapsulated in autophagosomes was facilitated by concurrent loading.

In this study, muscle fiber cross‐sectional area and protein synthesis rate by puromycin incorporation were not evaluated. Whether the changes in mTORC1 signaling activity due to concurrent loading of SA‐induced functional overload and DEN correlate with the adaptations in muscle fiber cross‐sectional area and protein synthesis should be investigated. Also, male mice were employed as experimental animals to exclude the effects on the estrous cycle on muscle protein metabolism and mass. Furthermore, the increased inflammatory response, oxidative stress, and myokine secretion associated with concurrent loading of SA‐induced functional overload and DEN may affect the muscle phenotype. The contralateral side of the SA leg in Experiment 1 is the sham leg, whereas the contralateral side of SA leg in Experiment 2 is the DEN+SA leg, and the surgical intervention of the contralateral leg in the SA‐treated leg is different between the experiments. Therefore, it is difficult to directly compare the quantitative results of Experiments 1 and 2, even for the same SA alone treatment, and future research should examine the effects of concurrent loading of SA‐induced functional overload and DEN on muscle mass and function from a broader perspective. Additionally, in the experimental model employed in this study, the sham leg defects in DU and OL mice may have generated a bias as comparison. Due to the nature of the analysis method, comparisons with the sham leg of different animals are forced, as significant differences between groups are tested by multiple comparisons in the parameters for which an interaction was identified. Since this study was based on two‐way ANOVA with SA or DEN for one leg (factor 1) and SA or DEN for both legs (factor 2), comparisons between the only SA or DEN treated leg of OL or DU mice and the sham leg of UO mice are a statistical limitation.

## 5 CONCLUSION

In summary, our data showed that (1) DEN attenuated the functional overload‐induced mTORC1 signaling activation and muscle hypertrophy, and (2) functional overload partially improved the DEN‐induced increase in muscle protein degradation and atrophy. These findings suggest that functional overload‐induced muscle hypertrophy or DEN‐induced muscle atrophy was suppressed by concurrent loading of both surgical procedures. The results of this study may present important insights to achieve an understanding of the molecular responses governing the quantitative plasticity of skeletal muscle.

## AUTHOR CONTRIBUTIONS

All authors conceived and designed the project. Kazuki Uemichi and Takanaga Shirai performed the experiments. Kazuki Uemich analyzed the data. All authors interpreted the results of experiments. Kazuki Uemich prepared the figures and drafted the manuscript. Kazuki Uemich and Tohru Takemasa made manuscript revisions. All authors have read and approved the final manuscript and agree to be accountable for all aspects of the work and ensuring that questions related to the accuracy or integrity of any part of the work are appropriately investigated and resolved. All persons designated as authors qualify for authorship, and all those who qualify for authorship are listed.

## CONFLICT OF INTEREST STATEMENT

The authors declare that there are no conflicts of interest.

## ETHICS STATEMENT

All experimental procedures performed in this study were approved by the Institutional Animal Experiment Committee of the University of Tsukuba (animal ethical approval number: 21‐374) and were performed in accordance with the guidelines of the National Institutes of Health for the Care and Use of Laboratory Animals. All experiments conformed to the principles and standards of reporting in animal experiments of Experimental Physiology (Grundy, 2015).

## Supporting information


Figure S3:

Figure S4:

Figure S5:

Figure S6:
