# Supplementary material for: Combined effects of functional overload and denervation on skeletal muscle mass and its regulatory proteins in mice
Source: Physiol Rep. 2023 May 9;11(9):e15689. doi: 10.14814/phy2.15689 (PMC10169777; doi:10.14814/phy2.15689)

(a) Phos-Akt (Thr308)

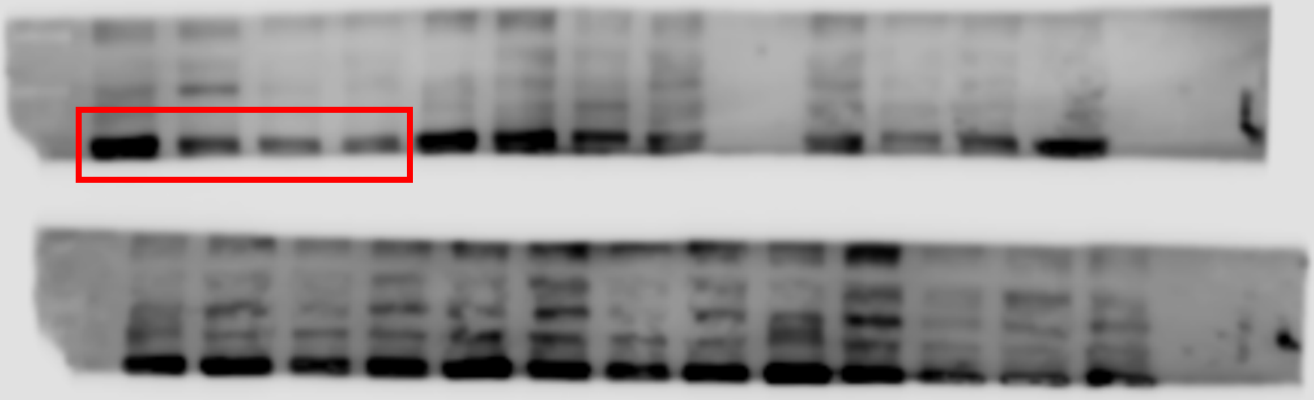

(b) Phos-Akt (Ser473)

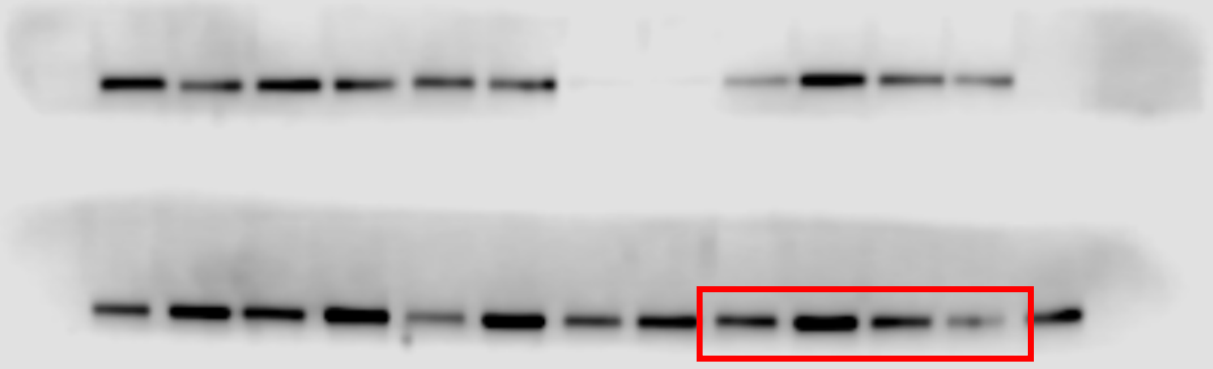

(C) Total Akt

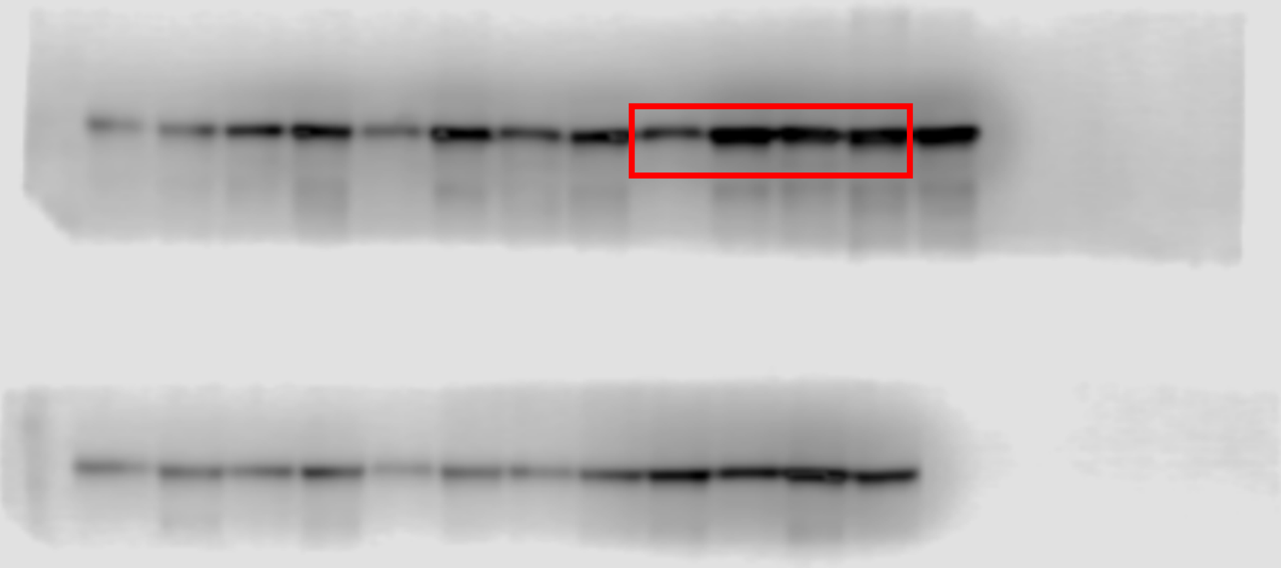

(d) Phos-P70S6K (Thr389)

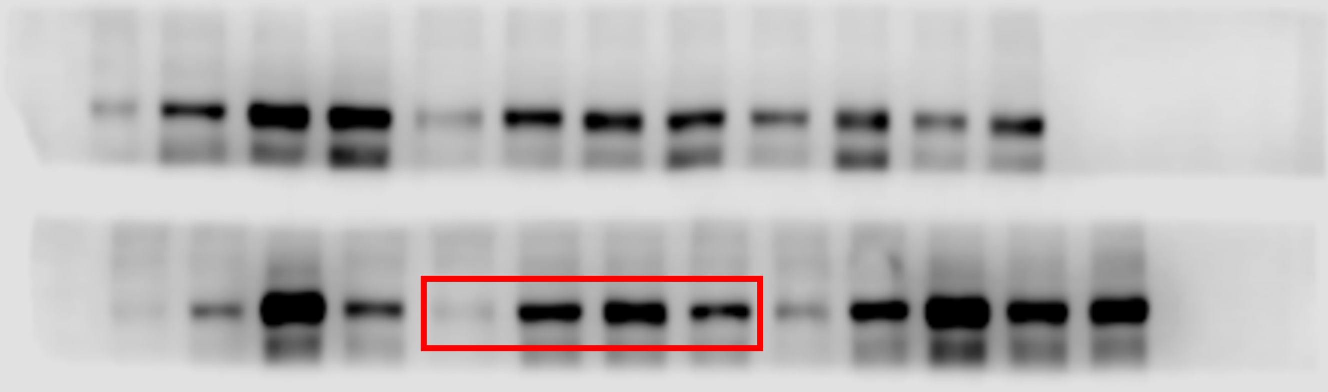

(e) Phos-P70S6K (Thr421/Ser424)

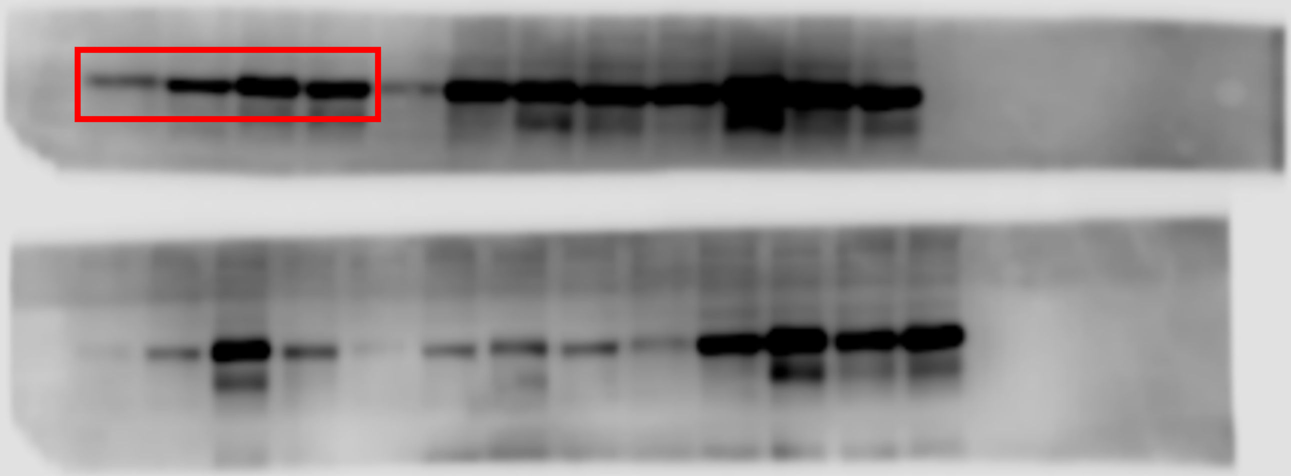

(f) Total P70S6K

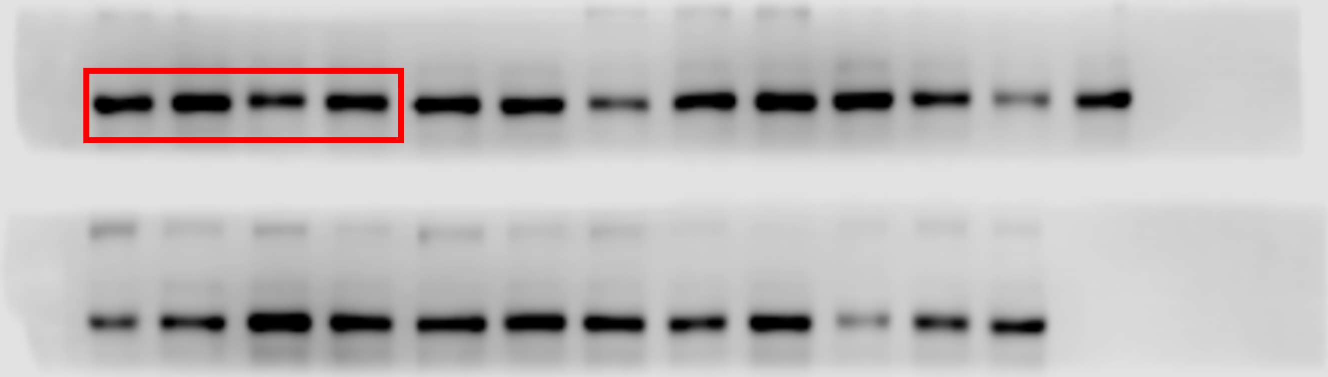

(g) Phos-Akt (Thr308)

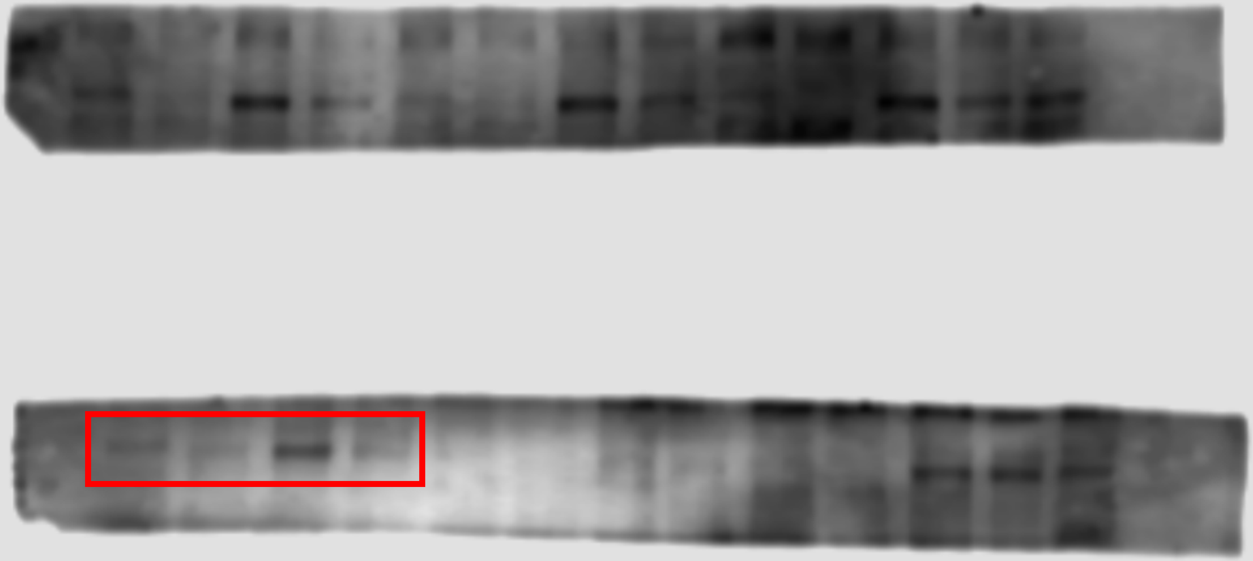

(h) Phos-Akt (Ser473)

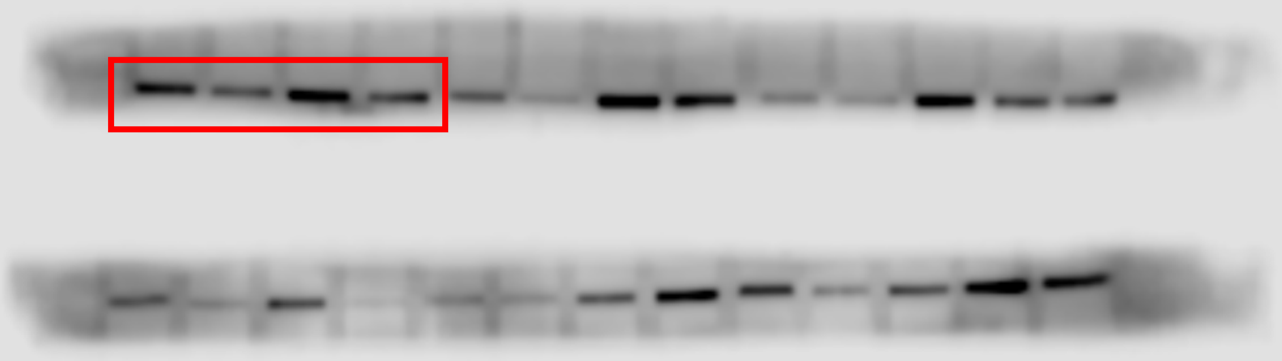

(i) Total Akt

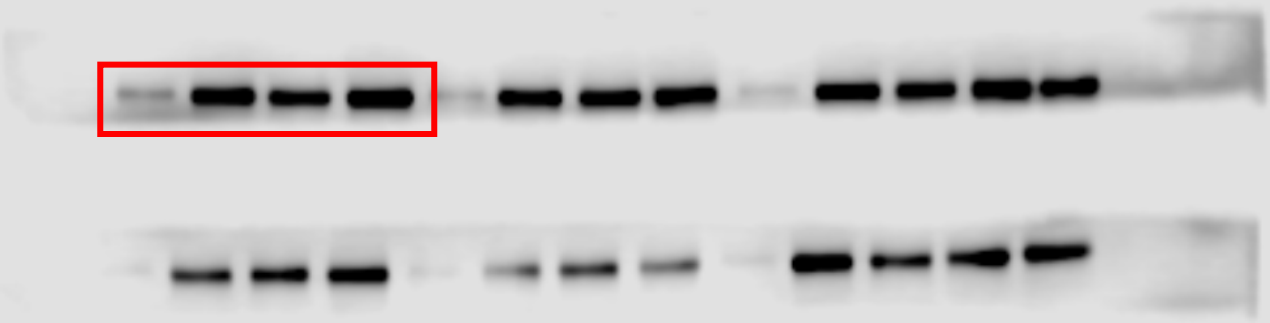

(j) Phos-P70S6K (Thr389)

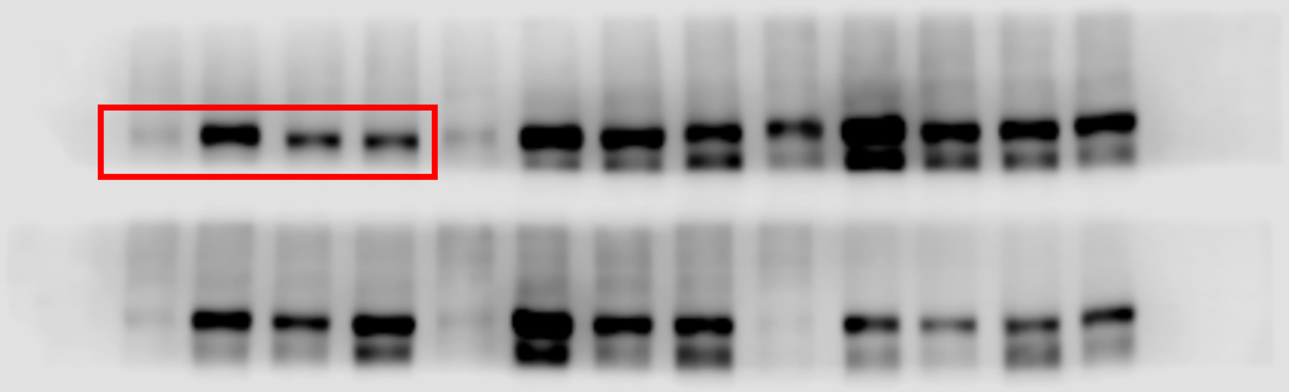

(k) Phos-P70S6K (Thr421/Ser424)

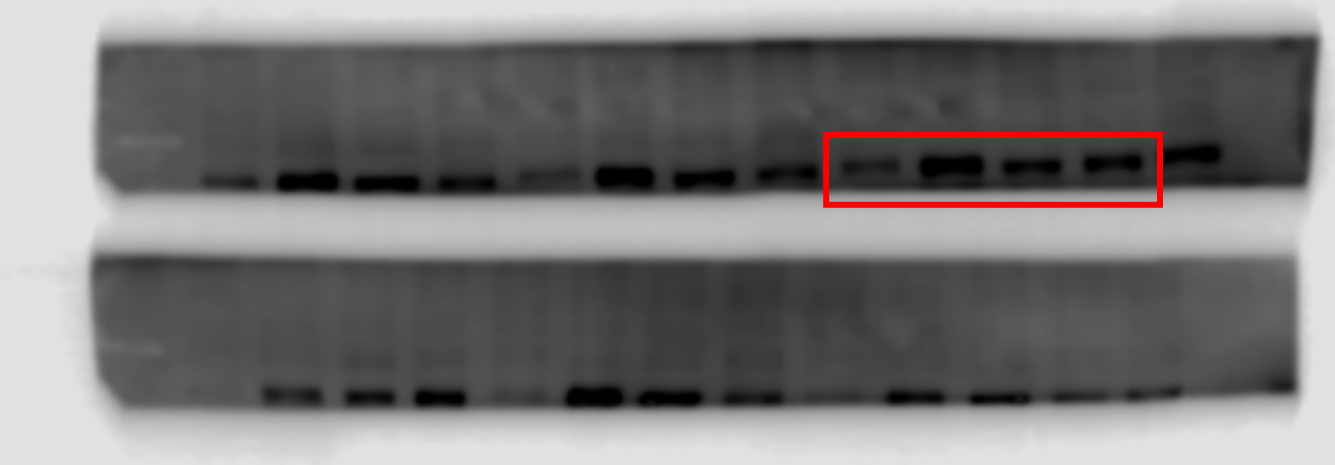

(l) Total P70S6K

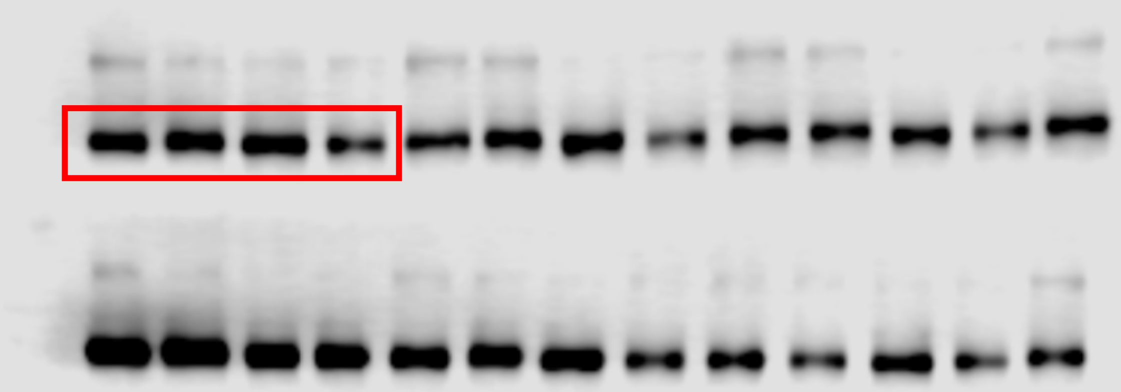

Supplement: Supplementary file 1 — Figure S3: [file PHY2-11-e15689-s003.pdf]
