# Supplementary material for: Combined effects of functional overload and denervation on skeletal muscle mass and its regulatory proteins in mice
Source: Physiol Rep. 2023 May 9;11(9):e15689. doi: 10.14814/phy2.15689 (PMC10169777; doi:10.14814/phy2.15689)

(a) Phos-RpS6 (Ser240/244)

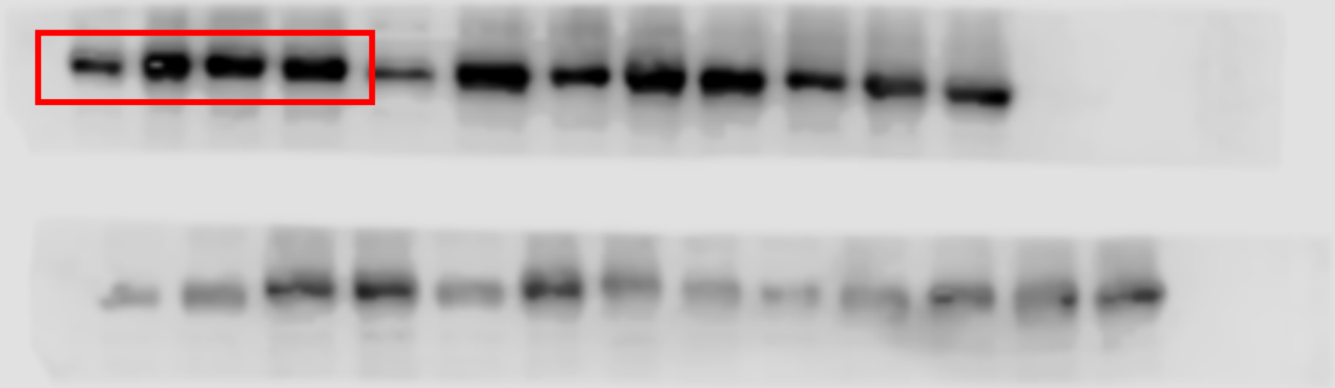

(b) Phos-RpS6 (Ser235/236)

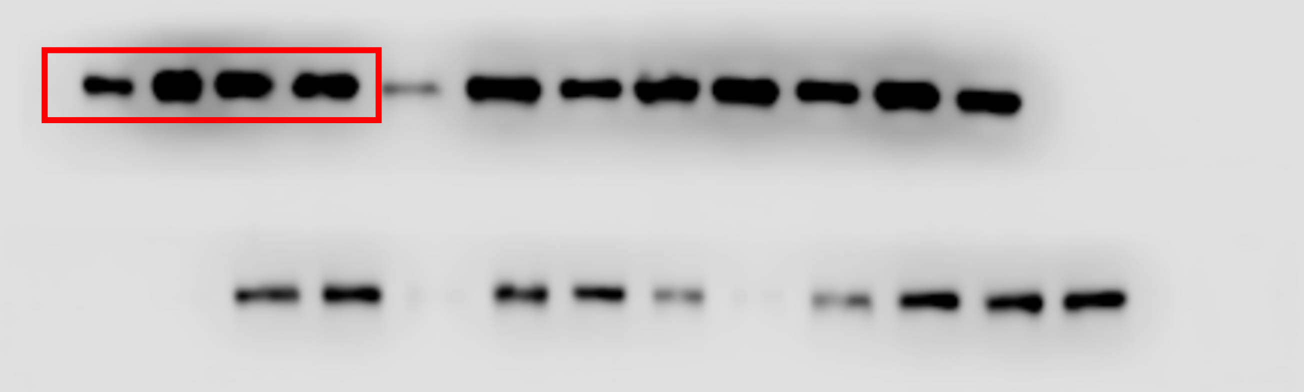

(c) Total RpS6

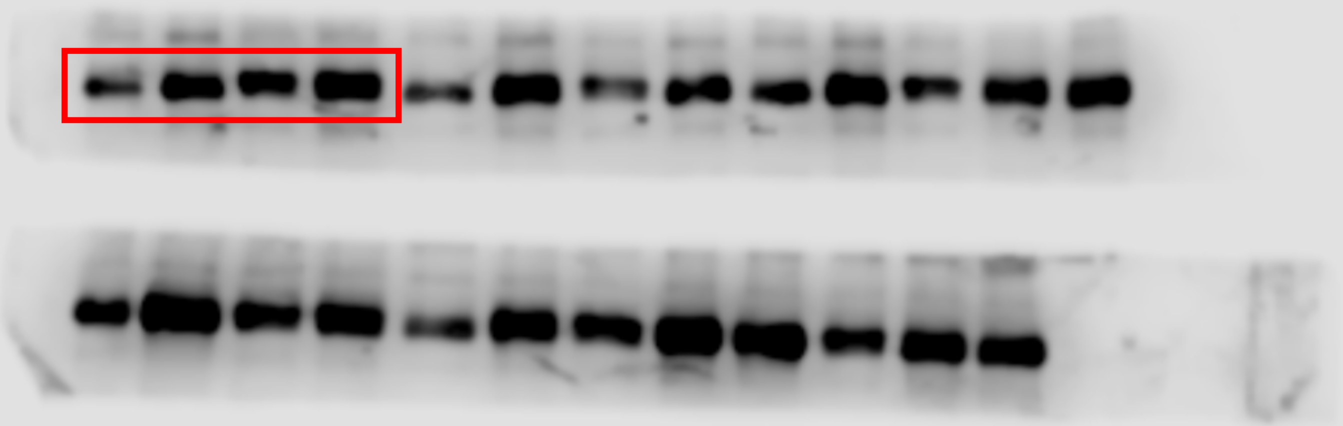

(d) Phos-4EBP1 (Thr37/46)

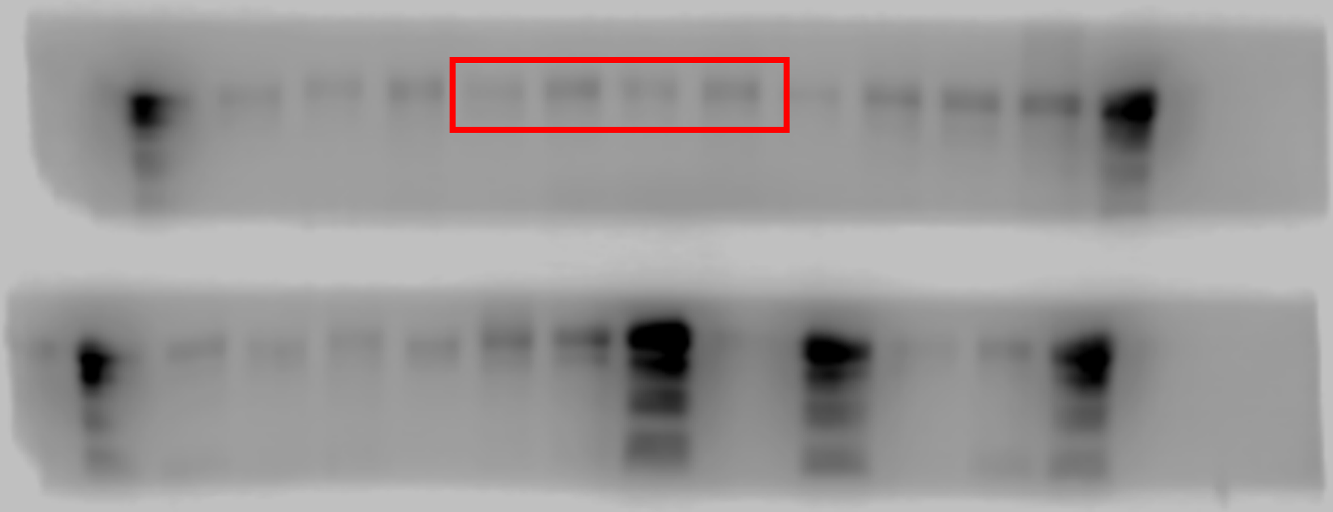

(e) Phos-4EBP1 (Ser65)

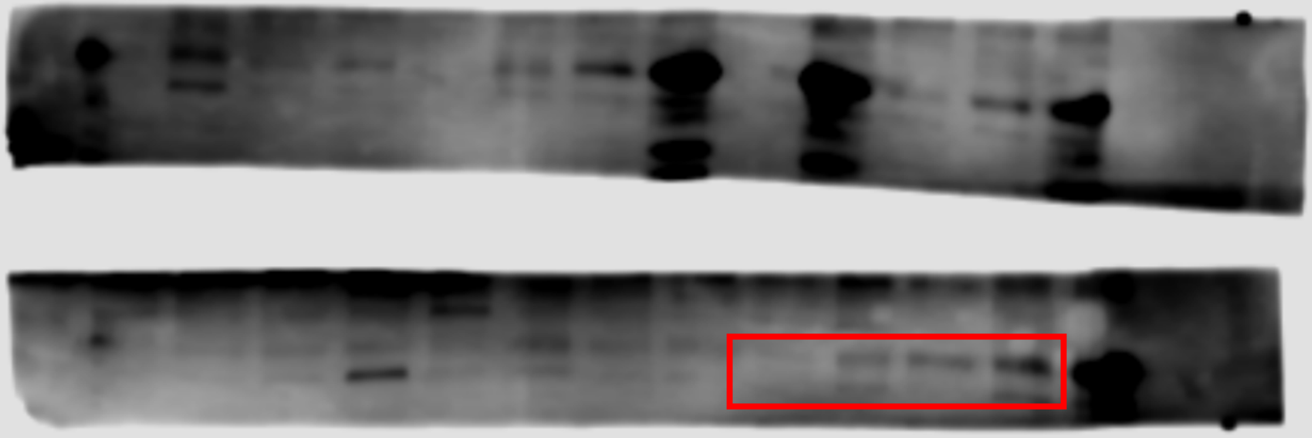

(f) Total 4EBP1

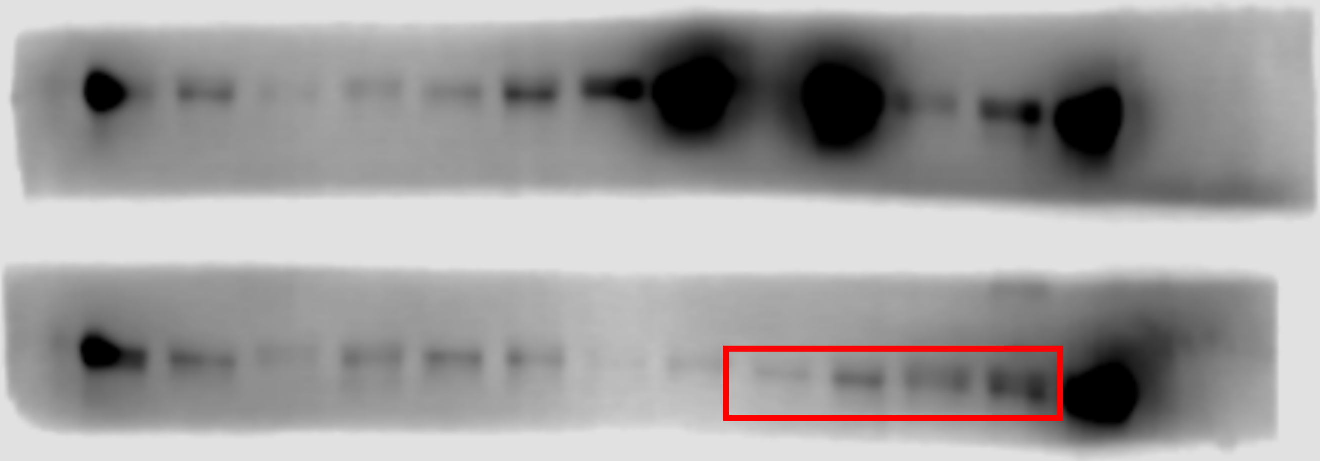

(g) Phos-RpS6 (Ser240/244)

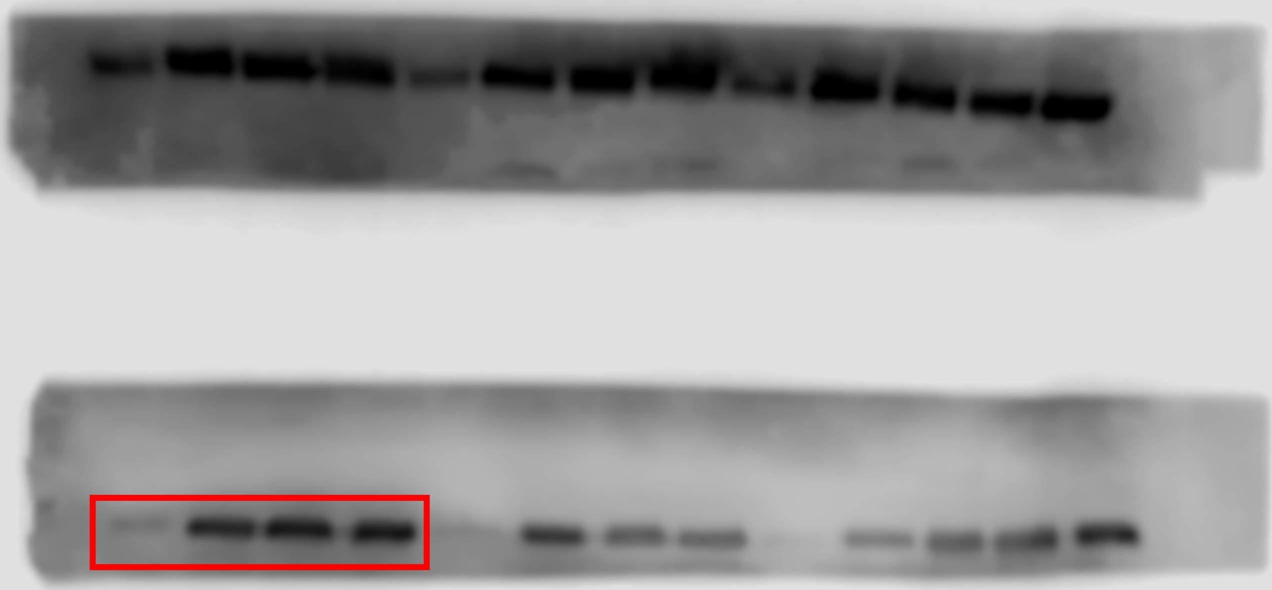

(h) Phos-RpS6 (Ser235/236)

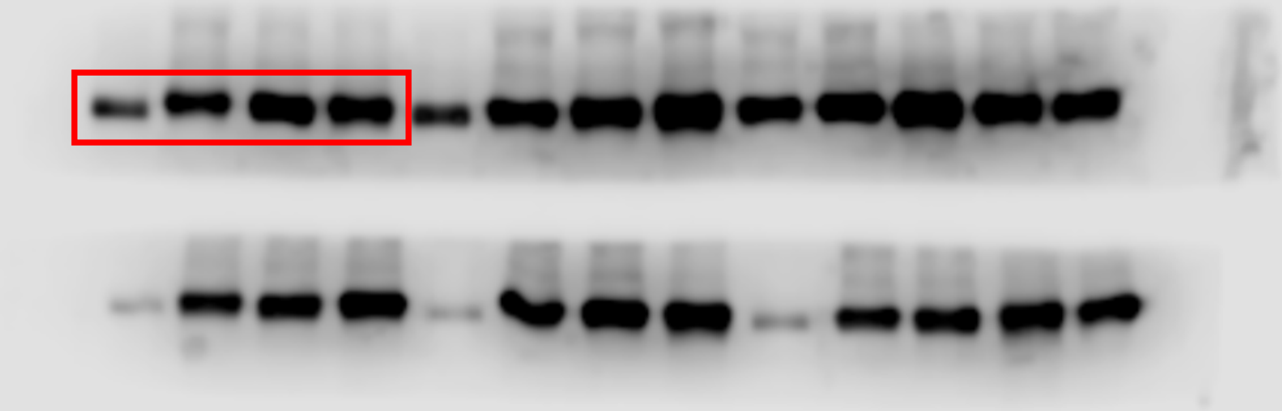

(i) Total RpS6

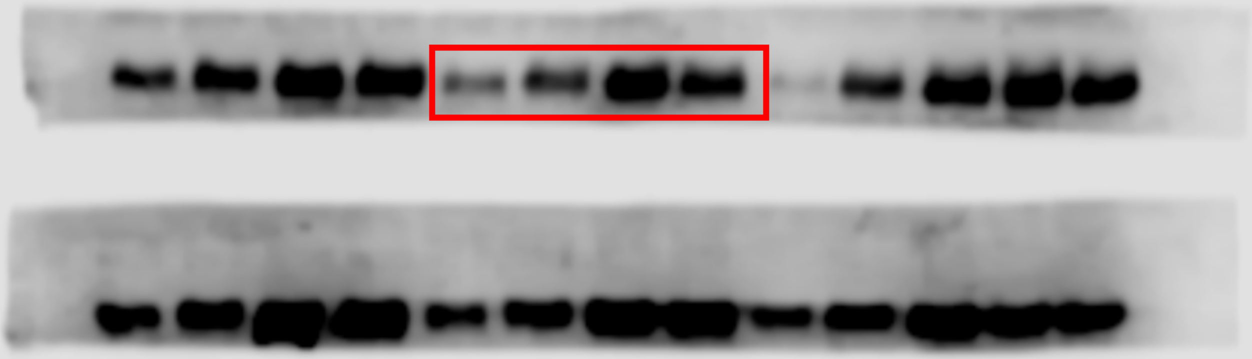

(j) p-4EBP1 (Thr37/46)

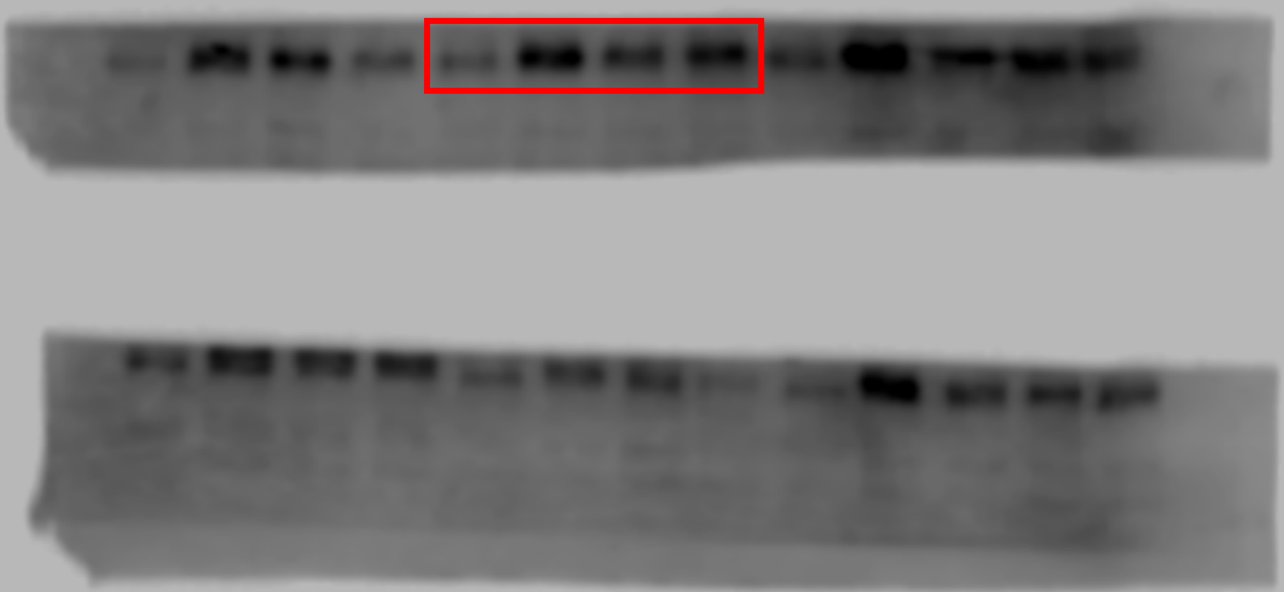

(k) Phos-4EBP1 (Ser65)

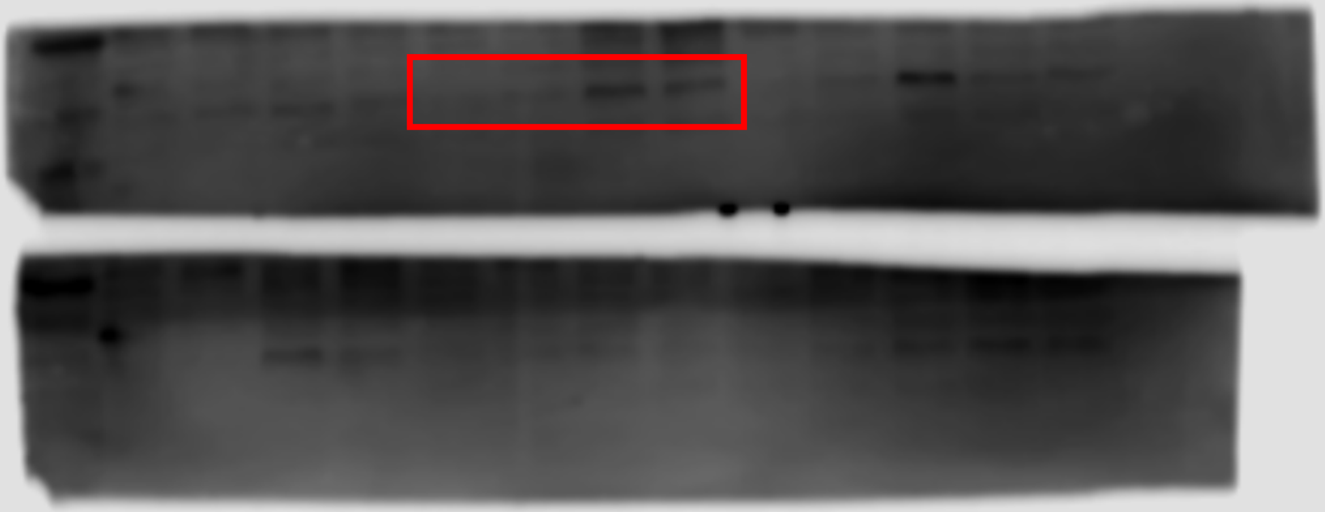

(l) Total 4EBP1

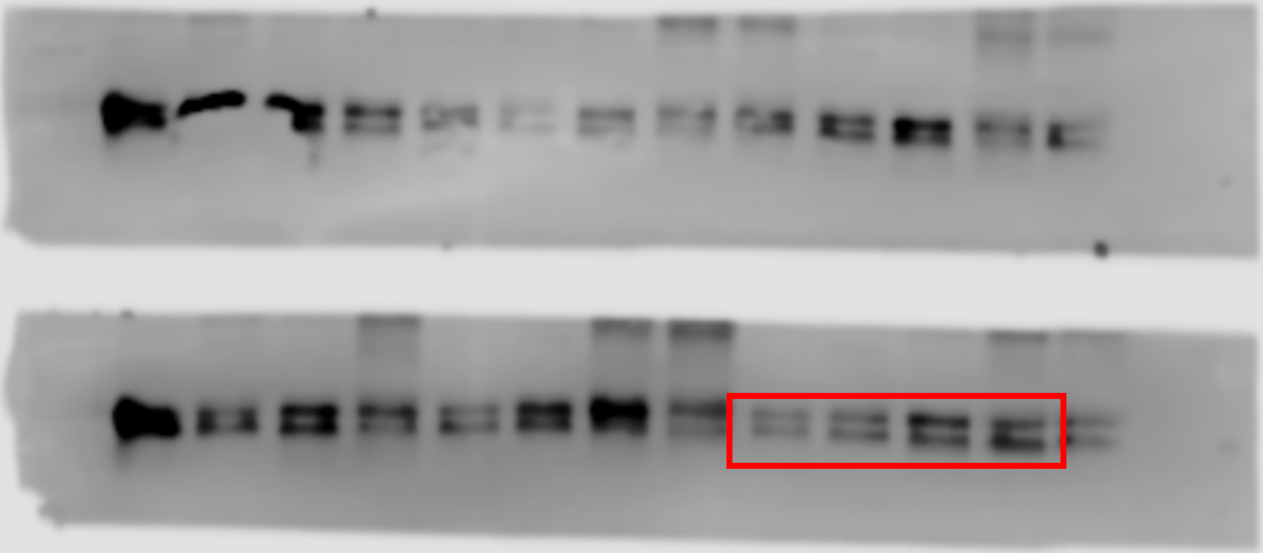

Supplement: Supplementary file 2 — Figure S4: [file PHY2-11-e15689-s004.pdf]
