# Supplementary figures and images for: Combined effects of functional overload and denervation on skeletal muscle mass and its regulatory proteins in mice
Source: Physiol Rep. 2023 May 9;11(9):e15689. doi: 10.14814/phy2.15689 (PMC10169777; doi:10.14814/phy2.15689)

**(a,b,c) LC3**

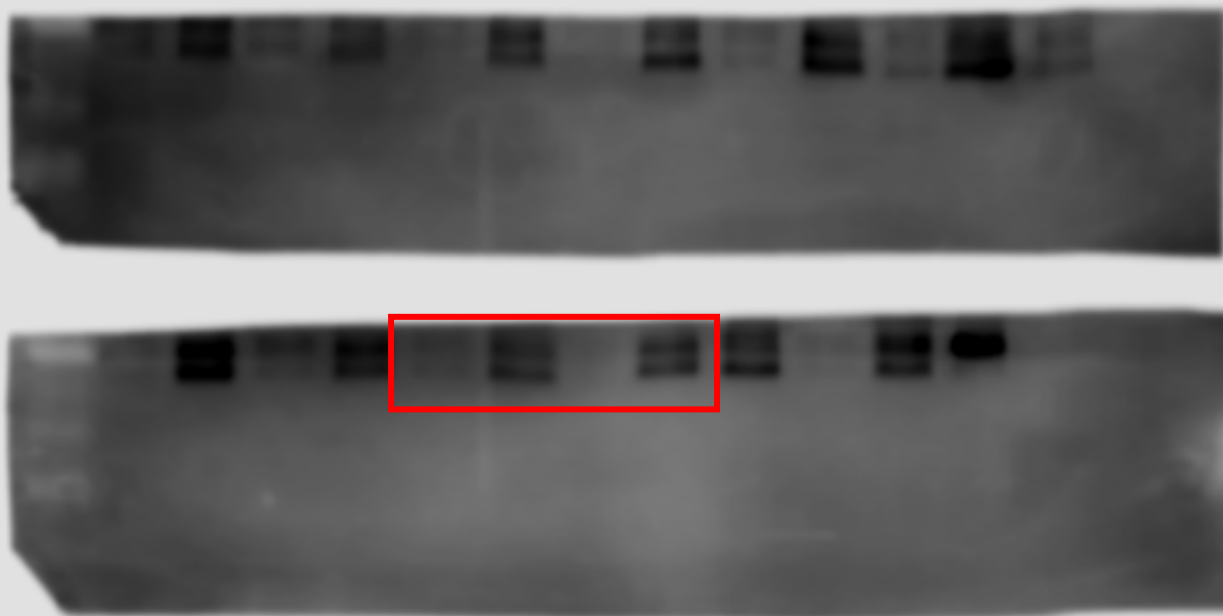

**(d) p62/SQSTM1**

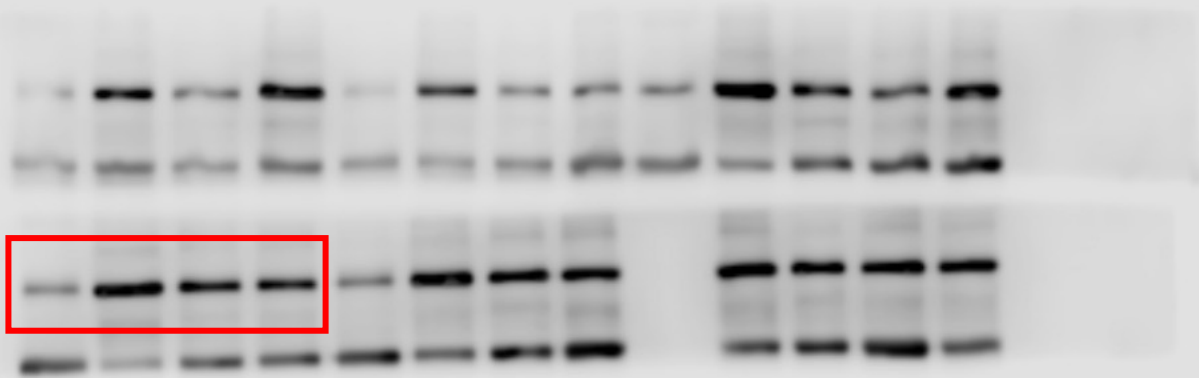

**(e,f,g) LC3**

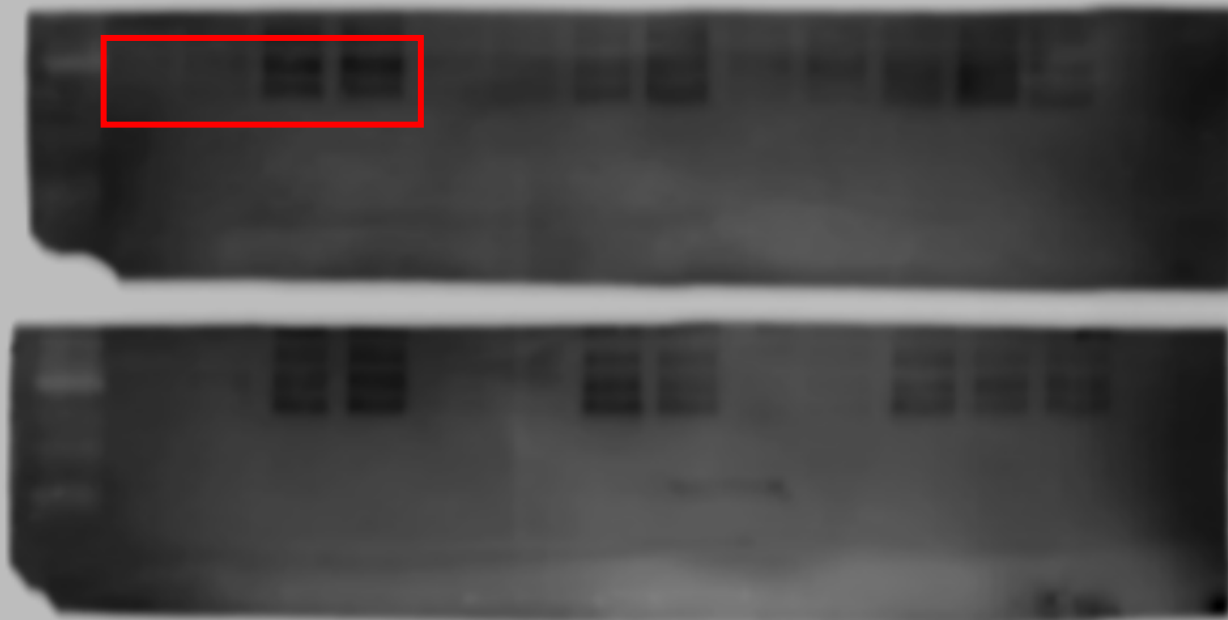

**(h) p62/SQSTM1**

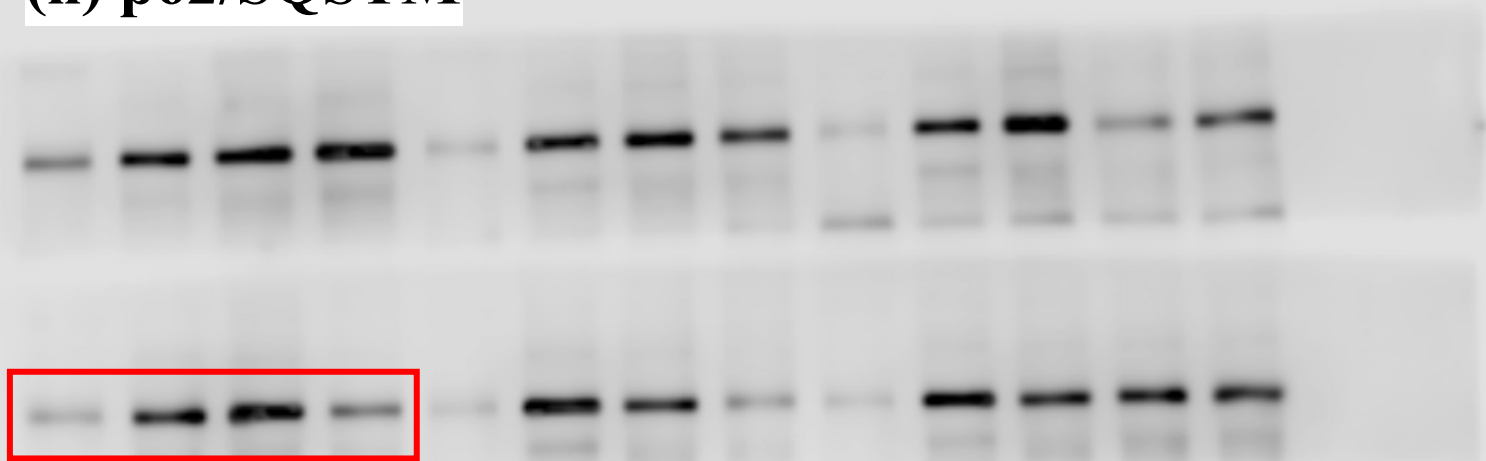

Supplement: Supplementary file 3 — Figure S5: [file PHY2-11-e15689-s001.pdf]
