# Supplementary material for: Combined effects of functional overload and denervation on skeletal muscle mass and its regulatory proteins in mice
Source: Physiol Rep. 2023 May 9;11(9):e15689. doi: 10.14814/phy2.15689 (PMC10169777; doi:10.14814/phy2.15689)

(a) Phos-FoxO1 (Ser256)

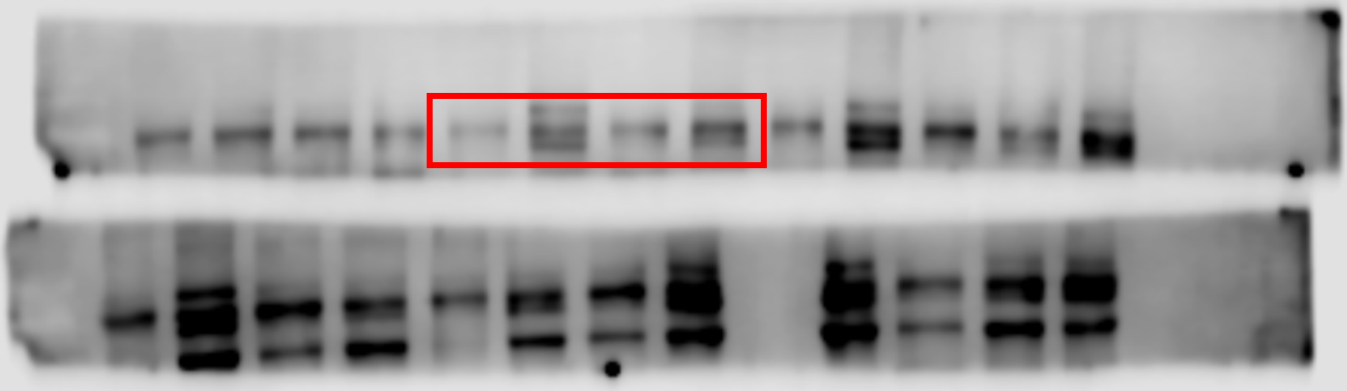

(d) Ubiquitinated proteins

(b) Total FoxO1

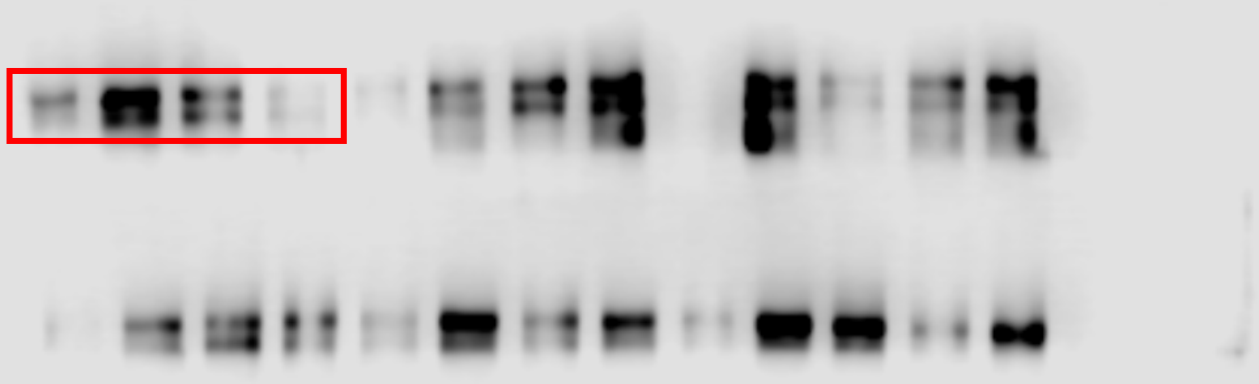

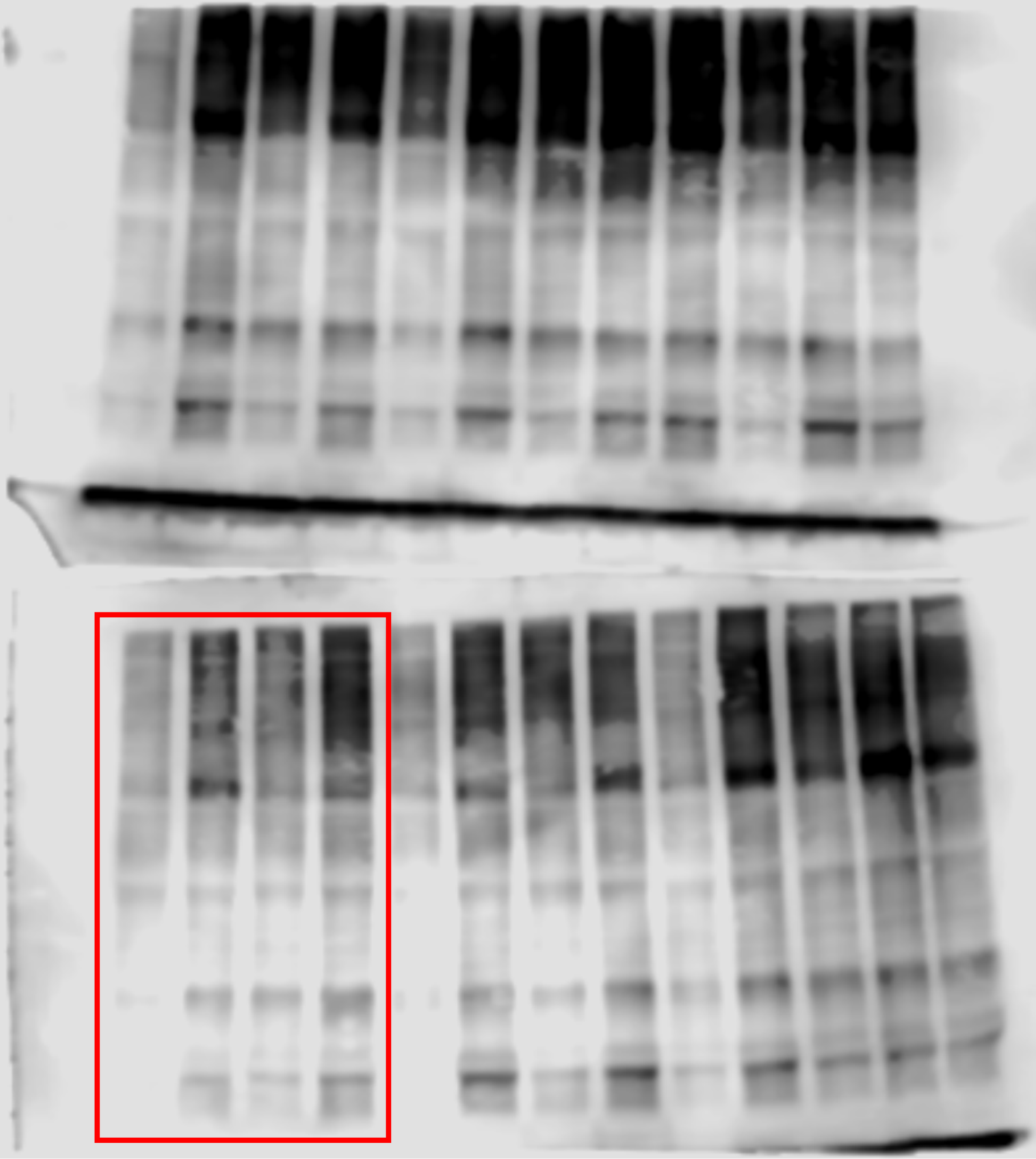

(e) Phos-FoxO1 (Ser256)

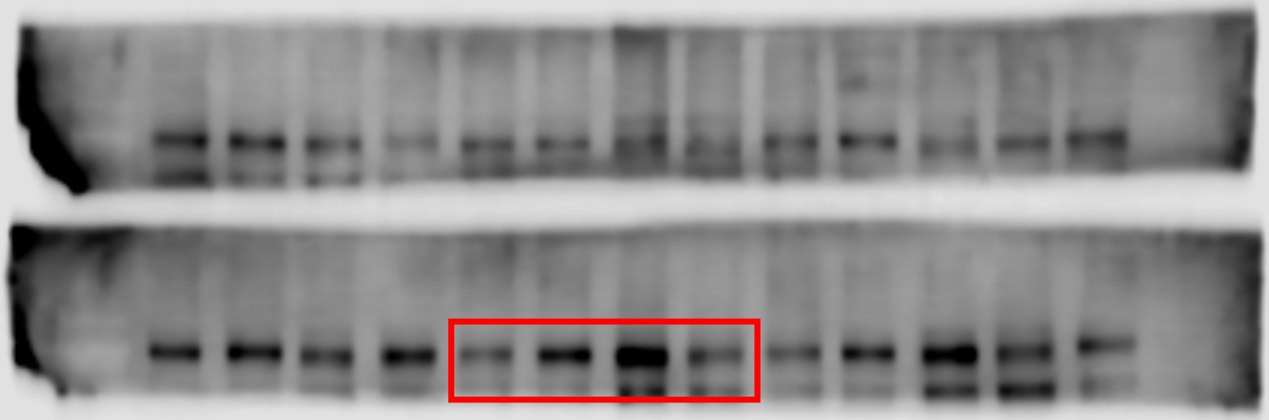

(f) Total FoxO1

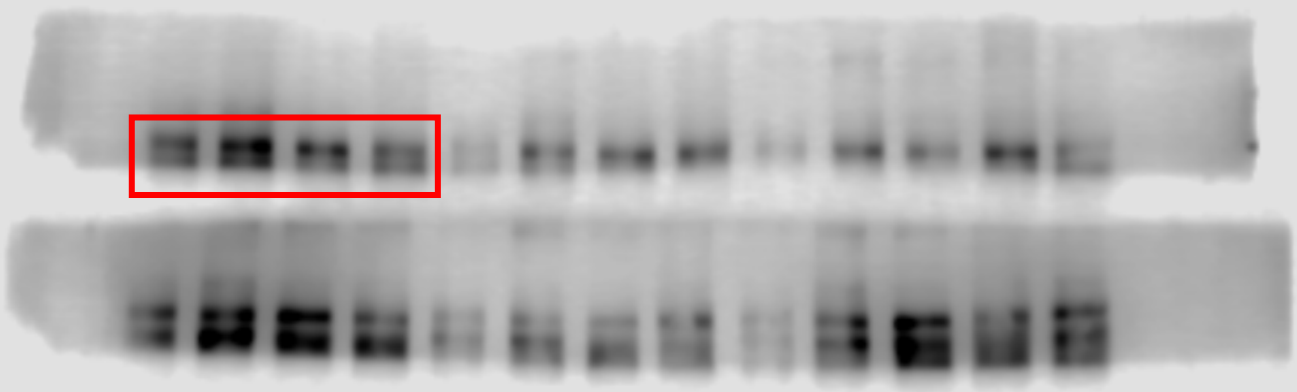

(g) Ubiquitinated proteins

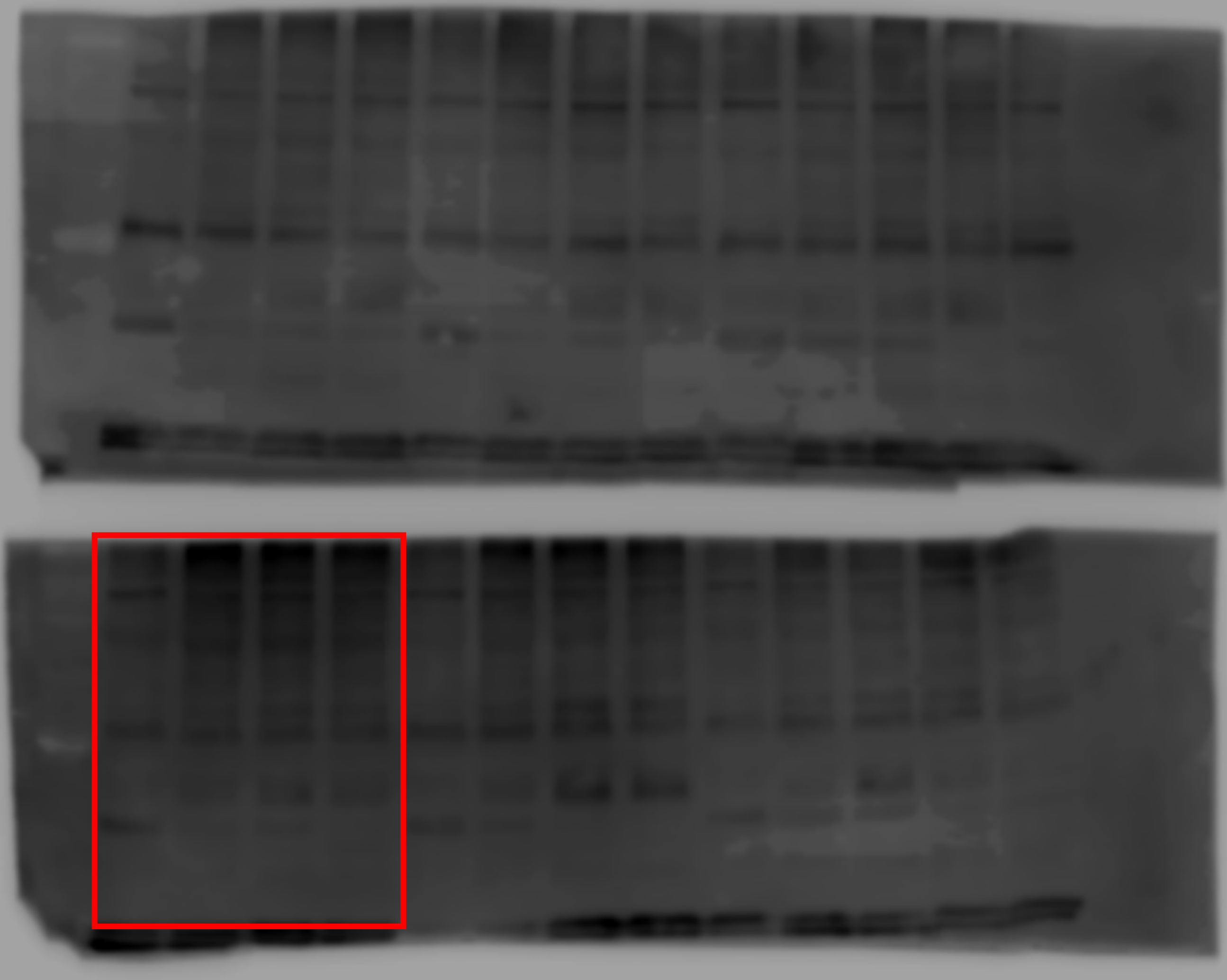

Supplement: Supplementary file 4 — Figure S6: [file PHY2-11-e15689-s002.pdf]
